# Genomic stratification beyond *Ras/B‐Raf* in colorectal liver metastasis patients treated with hepatic arterial infusion

**DOI:** 10.1002/cam4.2415

**Published:** 2019-09-10

**Authors:** J. Joshua Smith, Walid K. Chatila, Francisco Sanchez‐Vega, Jashodeep Datta, Louise C. Connell, Bryan C. Szeglin, Azfar Basunia, Taryn M. Boucher, Haley Hauser, Isaac Wasserman, Chao Wu, Andrea Cercek, Jaclyn F. Hechtman, Chris Madden, William R. Jarnagin, Julio Garcia‐Aguilar, Michael I. D'Angelica, Rona Yaeger, Nikolaus Schultz, Nancy E. Kemeny

**Affiliations:** ^1^ Department of Surgery, Colorectal Service Memorial Sloan Kettering Cancer Center (MSKCC) New York City New York USA; ^2^ Human Oncology and Pathogenesis Program, MSKCC New York City New York USA; ^3^ Center for Molecular Oncology MSKCC New York City New York USA; ^4^ Tri-Institutional Program in Computational Biology & Medicine New York City New York USA; ^5^ Department of Surgery Hepatopancreatobiliary Service, MSKCC New York City New York USA; ^6^ Department of Medicine MSKCC New York City New York USA; ^7^ Department of Pathology MSKCC New York City New York USA; ^8^ Department of Epidemiology & Biostatistics MSKCC New York City New York USA

**Keywords:** colorectal cancer, floxuridine, implantable infusion pumps, liver, metastasis

## Abstract

**Background:**

Resection of colorectal liver metastases (CLM) can cure disease, but many patients with extensive disease cannot be fully resected and others recur following surgery. Hepatic arterial infusion (HAI) chemotherapy can convert extensive liver disease to a resectable state or decrease recurrence risk, but response varies and no biomarkers currently exist to identify patients most likely to benefit.

**Methods:**

We performed a retrospective cohort study of CLM patients receiving HAI chemotherapy whose tumors underwent MSK‐IMPACT sequencing. The frequency of oncogenic alterations and their association with overall survival (OS) and objective response rate were analyzed at the individual gene and signaling pathway levels.

**Results:**

Three hundred and seventy patients met inclusion criteria: 189 (51.1%) who underwent colorectal liver metastasectomy followed by HAI + systemic therapy (Adjuvant cohort), and 181 (48.9%) with unresectable CLM (Metastatic cohort) who received HAI + systemic therapy, consisting of 63 (34.8%) with extrahepatic disease and 118 (65.2%) with liver‐restricted disease. Genomic alterations were similar in each cohort, and no individual gene or pathway was significantly associated with objective response. Patients in the adjuvant cohort with concurrent *Ras/B‐Raf* alteration and *SMAD4* inactivation had worse prognosis while in the metastatic cohort patients with co‐alteration of *Ras/B‐Raf* and *TP53* had worse OS. Similar findings were observed in a validation cohort.

**Conclusions:**

Concurrently altered *Ras/B‐Raf* and *SMAD4* mutations were associated with worse survival in resectable patients, while concurrent *Ras/B‐Raf* and *TP53* alterations were associated with worse survival in unresectable patients. The mutual exclusivity of *Ras/B‐Raf*, *SMAD4*, and *TP53* may have prognostic value for CLM patients receiving HAI.

## Graphical abstract

Patients with colorectal liver metastases receiving hepatic arterial infusion chemotherapy who have either resectable disease with concurrently altered Ras/B‐Raf and SMAD4 mutations, or unresectable disease with concurrent Ras/B‐Raf and TP53 alterations had worse survival. Mutual exclusivity of Ras/B‐Raf, SMAD4, and TP53 status may be useful for prognostic stratification in these patients.

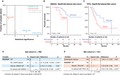

## 1 INTRODUCTION

Colorectal cancer (CRC) is the third most common cancer in the United States, accounting for an estimated 140 000 new cases and 50 000 deaths annually. The liver is the most common metastatic site for CRC, with 60% of CRC patients developing colorectal liver metastases (CLM).^1^ Patients with limited CLM can achieve long‐term survival if all disease can be removed surgically or ablated.^2^, ^3^ Recently, genomic biomarkers that correlate with outcomes in patients with CLM undergoing resection have been identified. For instance, RAS activating mutations are associated with shortened recurrence‐free survival and increased risk of extrahepatic recurrence after CLM resection.^4^ In addition, co‐occurrence of RAS and TP53 mutations is associated with shorter overall survival (OS).^5^ The presence of BRAF mutation is also a poor prognostic marker associated with higher risk of recurrence.^6^, ^7^, ^8^

While some patients with limited CLM can be cured, many CRC patients have liver metastases too extensive for resection or recur after surgery. Hepatic arterial infusion (HAI) chemotherapy provides high‐dose, liver‐directed chemotherapy that can convert unresectable CLM to a resectable state or decrease risk of recurrence in patients undergoing CLM resection.^9^ HAI has been associated with improved survival in CLM patients,^10^ but patients derive variable benefit; a subset achieve a pathologic complete response to treatment while other patients demonstrate disease progression within the liver despite best available current therapy. To date, there are no clinically applicable predictive biomarkers to select patients most likely to benefit from HAI.

In this study, we sought to characterize the mutational profiles of CLM patients treated with liver‐directed and systemic chemotherapy to investigate the correlation between genomic alterations and clinical outcome. We employed the FDA‐approved, comprehensive Next Generation Sequencing (NGS) assay, Memorial Sloan Kettering‐Integrated Mutation Profiling of Actionable Cancer Targets (MSK‐IMPACT),^11^ to identify genomic correlates to OS in patients receiving liver‐directed and systemic therapy.

## 2 MATERIALS AND METHODS

### 2.1 Patient selection

A waiver was obtained from the Institutional Review Board at Memorial Sloan Kettering Cancer Center (MSK) to identify CLM patients treated with HAI chemotherapy who also underwent tumor MSK‐IMPACT genomic testing (April 2015 and September 2016). Three hundred and seventy CLM patients receiving HAI chemotherapy met inclusion criteria and were included in a prospectively maintained clinical registry. Patients initiated HAI + systemic chemotherapy between October 1993 and August 2017; all patients received treatment at MSK. This is, to our knowledge, the largest series of HAI‐treated patients with combined molecular and clinical outcome data. HAI‐floxuridine (HAI‐FUDR) chemotherapy with dexamethasone (to limit biliary toxicity) was delivered over a 14‐day continuous infusion through HAI pumps, as described^12^, ^13^, ^14^; HAI therapy was administered in a 4‐week cycle. Patients also received systemic chemotherapy (Table 1). Electronic medical records were reviewed for clinical and pathological data, including demographics, primary and metastatic tumor characteristics, disease progression, and survival (Table S1a‐b).

**Table 1:** Demographic and clinical characteristics

	OVERALL (n = 370)	Adj (n = 189)	Met, total (n = 181)	Met‐EHD(−) (n = 118)	Met‐EHD(+) (n = 63)
Sex					
Male	203 (55)	100 (53)	103 (57)	70 (59)	33 (52)
Female	167 (45)	89 (47)	78 (43)	48 (41)	30 (48)
Age, median (range, yrs)	53 (26‐78)	54 (29‐78)	52 (26‐78)	52 (26‐73)	52 (26‐78)
26‐50, n (%)	152 (41)	73 (38)	79 (43)	49 (38)	30 (48)
51‐74, n (%)	214 (58)	113 (60)	101 (56)	69 (62)	32 (49)
≥75, n (%)	4 (1)	3 (2)	1 (1)	0 (0)	1 (3)
Site of Primary, n (%)					
Left colon	194 (52)	104 (55)	90 (50)	60 (51)	30 (48)
Right colon	89 (24)	39 (21)	50 (27)	29 (25)	21 (33)
Rectum	87 (24)	46 (24)	41 (23)	29 (24)	12 (19)
Lymph node positive primary, n (%)					
Yes	254 (68)	123 (65)	131 (72)	80 (68)	51 (81)
No	116 (32)	66 (35)	50 (28)	38 (32)	12 (19)
Synchronous disease, n (%)					
Yes	281 (76)	116 (61)	165 (91)	107 (91)	58 (92)
No	89 (24)	73 (39)	16 (9)	11 (9)	5 (8)
Systemic chemo prior to HAI^a^, n (%)					
Yes	322 (87)	166 (88)	156 (86)	102 (86)	54 (86)
No	48 (13)	23 (12)	25 (14)	16 (14)	9 (14)
First systemic chemo given with HAI, n (%)					
5‐FU/LV or Capecitabine	48 (13)	30 (16)	18 (10)	12 (10)	6 (10)
Irinotecan alone	46 (12)	16 (9)	30 (17)	19 (16)	11 (18)
Oxaliplatin/Irinotecan	30 (8)	0 (0)	30 (17)	21 (18)	9 (14)
FOLFIRI	133 (36)	60 (32)	73 (40)	49 (42)	24 (38)
FOLFOX	74 (20)	48 (25)	26 (13)	13 (11)	13 (20)
FOLFIRI + Anti‐EGFR	17 (5)	17 (9)	0 (0)	0 (0)	0 (0)
FOLFIRI or FOLFOX + Anti‐VEGF	7 (2)	6 (3)	1 (1)	1 (1)	0 (0)
None	15 (4)	12 (6)	3 (2)	3 (3)	0 (0)
EHD before HAI, n (%)					
Yes	77 (21)	14 (7)	63 (35)	0 (0)	63 (100)
No	293 (79)	175 (93)	118 (65)	118 (100)	0 (0)
Site of EHD before HAI^b^, n (%)					
Lung		12 (76)			20 (27)
Lymph node(s)		1 (6)			34 (47)
Peritoneum	‐	1 (6)	‐	‐	8 (11)
Ovary		1 (6)			6 (8)
Other		1 (6)			5 (7)
Hepatic progression of disease, n (%)					
Yes	174 (47)	59 (31)	115 (64)	75 (64)	40 (64)
No	196 (53)	130 (69)	66 (36)	43 (36)	23 (36)

Abbreviations: EHD: extrahepatic disease; chemo: chemotherapy; HAI: hepatic arterial infusion chemotherapy; VEGF: vascular endothelial growth factor; EGFR: epidermal growth factor receptor; 5‐FU/LV: 5‐fluorouracil/leucovorin; FOLFIRI: 5‐FU + leucovorin +irinotecan; FOLFOX: 5‐FU + leucovorin +oxaliplatin.

a Patients who received systemic chemotherapy prior to HAI.

b Patients who had extrahepatic disease in > 1 site.

Patients deemed resectable who underwent complete CLM resection followed by adjuvant HAI and systemic therapy comprised the Adjuvant cohort (Adj), while those with unresectable CLM who received HAI and systemic therapy comprised the Metastatic cohort (Met). In the Met cohort, objective responses by cross‐sectional imaging were determined^15^ and molecular correlates of response were examined.

A cohort of 93 patients, independent of the original 370 patients, and who had also undergone MSK‐IMPACT testing was identified at MSK for validation analyses and consisted of 43 Adj and 50 Met patients (Table S2). Finally, to determine if there were any differences in mutational profiles between cohorts undergoing and not undergoing HAI/systemic chemotherapy, an additional cohort of 317 CLM patients not treated with HAI and who underwent MSK‐IMPACT sequencing were evaluated.

### 2.2 Sample collection and next‐generation sequencing

Formalin‐fixed paraffin‐embedded (FFPE) tissue samples from patients' primary tumor or metastases were utilized for DNA extraction. Tumor‐specific genomic alterations were characterized using MSK‐IMPACT^11^; all patients signed consent to have their tumor DNA sequenced. MSK‐IMPACT is a clinically validated hybridization, capture‐based NGS assay that is performed in a CLIA accredited lab. The assay interrogates all exons and selects introns from over 400 cancer‐associated genes and assesses mutations, copy number changes, structural variants, and MSI status.^11^ Tumor and matched normal libraries were sequenced on an Illumina HiSeq 2500 (RRID: SCR_016383) and sequencing output was processed.^16^

MSK‐IMPACT was performed on the primary tumor in 122 patients (33%) and on the metastasis in 248 (67%); no significant differences were observed in the frequency of alterations between patients who had primary tumors vs metastatic tumors sequenced. A large majority of the sequenced metastases were liver metastases (202/248, 81%), with a number of lung metastases (15/248, 6%), lymph nodes (8/248, 3%), peritoneum (5/248, 2%), soft tissue (4/248, 2%), and other anatomic sites occurring at lower frequencies. Only 3 of 189 Adj cases (1.6%) and 1 of 181 (0.5%) Met cases were noted to be MSI‐high.

Genomic alterations were filtered for known driver variants using a priori knowledge via OncoKB (RRID: SCR_014782),^17^ as well as statistically recurrent hotspots^18^ and 3D hotspots.^19^ All other variants were excluded when defining mutant cases. Microsatellite Instability High (MSI‐High) samples were identified using the MSIsensor algorithm (RRID: SCR_006418)^20^ with a cut‐off score of 10.^21^

### 2.3 Statistical analysis

Two‐tailed t‐tests were used to compare continuous variables; Fisher's exact test was used to compare categorical variables. OS was defined as the interval from initiation of HAI‐FUDR to date of death or last follow‐up. The association between OS and somatic gene alterations was assessed for each cohort by examining separately every gene with *at least* five altered cases within the subgroup being examined. Progression‐free survival (PFS) was used in the Met validation cohort and was defined as any progression of disease in the liver or other sites after initiation of liver‐directed therapy. The log‐rank test was used to ascertain OS or PFS differences between gene‐wild type and gene‐altered patients. Multiple hypothesis correction was performed using the Benjamini‐Hochberg method, and q‐values ≤ 0.1 were considered to be significant. All statistical analyses were performed using R (RRID: SCR_001905) and SAS v9.4 (Cary, NC; RRID: SCR_008567).

## 3 RESULTS

### 3.1 Demographics and clinical characteristics of the study cohort

**Figure 1:**
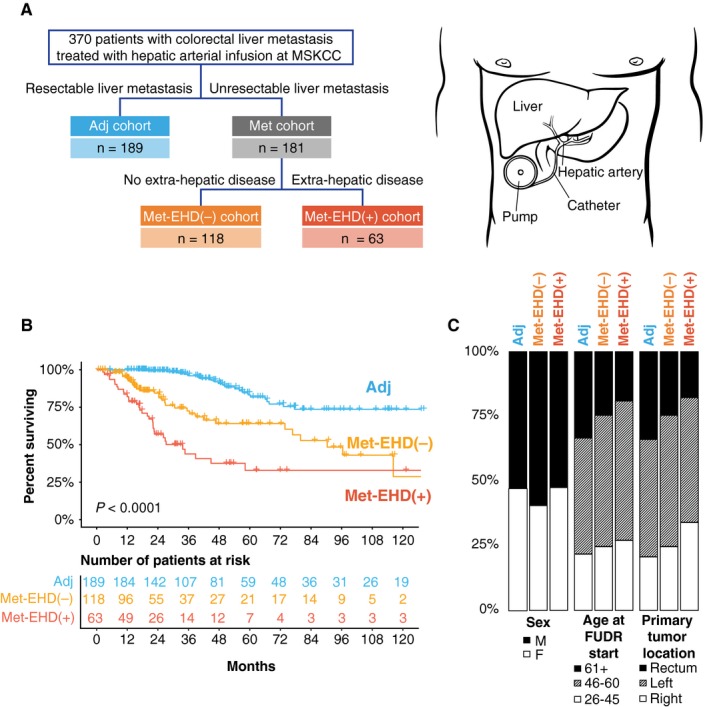
Study flow and basic clinical characteristics. (A), Patient selection flow diagram. Patients were stratified into resectable patients receiving adjuvant hepatic arterial infusion (HAI, Adjuvant cohort [Adj]) or unresectable (Metastatic cohort [Met]) patients with either extrahepatic disease (Met‐EHD[+]) or liver‐only disease (Met‐EHD[−]); (B), Kaplan‐Meier analysis of overall survival (OS) from initiation of HAI chemotherapy in resected patients, stratified by the Adj, Met‐EHD(+) and Met‐EHD(−) categories. (C), Comparison of clinical features across the three categories; Adj, Met‐EHD(+), Met‐EHD(−). Comparison of somatic alteration frequencies in recurrently altered genes between the three HAI cohorts

The analysis included 370 patients with histologically confirmed CLM treated with HAI‐FUDR and systemic therapy, with 189 (51.1%) and 181 (48.9%) patients in the Adj and Met cohorts, respectively (Table 1, Figure 1A). Of the 181 Met patients, 118 (65.2%) had liver‐restricted disease (extrahepatic disease‐negative, Met‐EHD[‐]), while 63 patients (34.8%) had concomitant liver and extrahepatic disease‐positive (Met‐EHD[+]; Figure 1A). Extrahepatic sites of disease included distant lymph nodes (34/63, 54%), lung (18/63, 28.6%), peritoneum (8/63, 12.7%), ovary (7/63, 11.1%), umbilicus (2/63, 3.1%), adrenal gland (1/63, 1.6%), omentum (1/63, 1.6%), and spleen (1/63, 1.6%). Median follow‐up after HAI chemotherapy initiation was 30.6 months (range, 0.1‐279.6 months). Expectedly, patients who underwent complete CLM resection and Adj HAI demonstrated the longest median OS, followed by patients with unresectable CLM and Met‐EHD(−) disease, with the Met‐EHD(+) cohort demonstrating relatively poor median OS (not reached [NR] vs 90.3 vs 33.7 months, respectively; *P* < .001; Figure 1B).

Demographic and treatment‐related clinical and pathologic characteristics of all patients are presented in Figure 1C and Table 1. No significant differences in outcomes were noted between Met and Adj cohorts based on systemic chemotherapy regimens delivered, except in the Met‐EHD(−) cohort wherein patients receiving systemic FOLFIRI demonstrated worse OS post‐HAI vs 5‐FU/FOLFOX based regimens (Figure S1).

### 3.2 Differences in mutational profiles in adjuvant and metastatic HAI subsets

**Figure 2:**
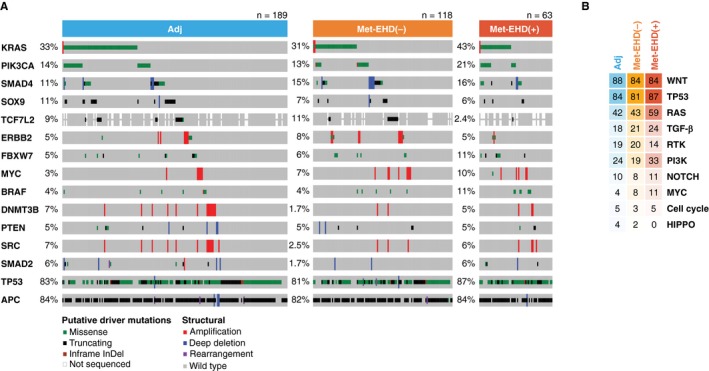
Genomic profile of each cohort. (A), OncoPrint representation of the 15 most frequently altered genes with types of gene alteration grouped by driver mutation or structural alterations in Adj, Met‐EHD(−), and Met‐EHD(+) cohorts. (B), Frequencies of alterations across 10 canonical signaling pathways across the 3 cohorts

In comparing frequency of somatic alterations in HAI (n = 370) vs non‐HAI (n = 317) CLM patients, *KRAS* and *BRAF* mutations were more frequent in the non‐HAI cohort (q = 0.001 for *KRAS*; q = 0.05 for *BRAF*; Figure S2), likely representing patient selection for liver‐limited disease in the HAI cohort.^22^ In contrast, no statistically significant differences in somatic alteration frequencies were observed for any individual gene across the Adj, Met‐EHD(‐), and Met‐EHD(+) subgroups (Figure 2A). *TP53* and *KRAS* alterations were mutually exclusive in both Adj (q = 0.002) and combined Met (q = 0.003) cohorts. In Adj patients, *TP53* alterations were mutually exclusive with *SOX9* truncating mutations (q = 0.068). Of note, no enrichment of canonical cell signaling pathway alterations (as defined in Sanchez‐Vega et al^23^) was observed across Adj, Met‐EHD(−), and Met‐EHD(+) cohorts (Figure 2B).

### 3.3 Association between individual gene and pathway‐level alterations, objective response, and overall survival

Examination of the relative contributions of individual gene alterations (Figure S3) and pathway‐level alterations to objective response following HAI chemotherapy in the Met cohort revealed a near‐significant association of cell cycle signaling (8% in responding patients vs 0% in nonresponding patients, q = 0.065), and no significant *RAS‐RTK*, *WNT/β‐catenin*, or *TP53* pathway alterations associated with response.

In previous work, we had reported the impact of *RAS/BRAF* signaling alterations on outcomes for HAI‐treated mCRC patients,^24^ which is consistent with the worse OS exhibited by *Ras/B‐Raf* altered patients across our cohort and subcohorts (Figure S4). Because of this correlation with outcomes, we used *Ras/B‐Raf* status (as determined by the presence or absence of known driver mutations in *BRAF*, *KRAS,* or *NRAS*) as a stratification criterion for all our subsequent analyses of associations between genomic alterations and OS.

**Figure 3:**
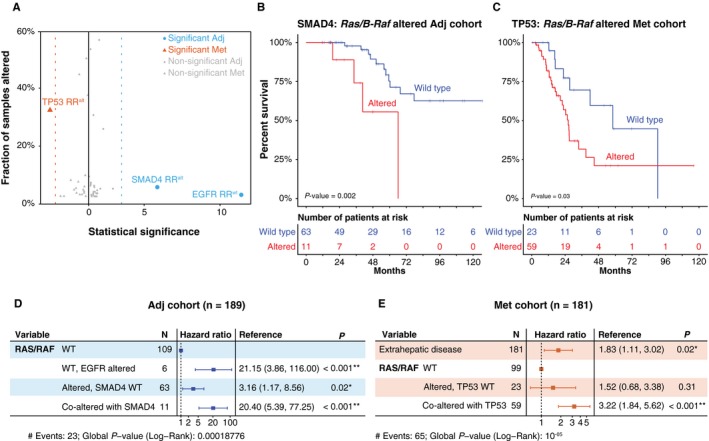
Associations between survival and gene alterations. (A), Analysis of associations between gene alterations and overall survival (OS), stratified by subcohort and *Ras/B‐Raf* alteration status. (B‐C), Kaplan‐Meier curves illustrating differences in OS for *SMAD4* and *TP53* relative to subcohort associations highlighted in (A). (D‐E), Results from multivariate analysis using Cox proportional‐hazards model

To determine if we could further inform prognosis beyond *Ras/B‐Raf* status, we examined survival of patients in the Adj cohort within *Ras/B‐Raf* altered (n = 63, 33%) and *Ras/B‐Raf* wild type (n = 126, 67%) subgroups. Similarly, we also examined the Met cohort according to *Ras/B‐Raf* altered (n = 82, 45%) and *Ras/B‐Raf* wild‐type (n = 99, 55%) status. The only genes significantly correlated with genomic events and survival were *SMAD4* and *EGFR* in the Adj cohort and *TP53* in the Met cohort (Figure 3A, Table S3a‐h). Specifically, within the Adj‐*Ras/B‐Raf* altered group, inactivation of *SMAD4* (n = 11) was associated with worse prognosis (Figure 3B, q = 0.01). Within the Adj‐*Ras/B‐Raf* wild‐type group, *EGFR* amplification (n = 6) was associated with decreased survival (Figure S5, q < 0.001). Furthermore, alterations in *TP53* were associated with worse survival in the subset of Met patients with concurrent *Ras/B‐Raf* alterations (Figure 3C, q = 0.15). Of note, we observed that *SMAD4* alterations were only associated with worse OS within the Adj group when they co‐occurred with *Ras/B‐Raf* alterations but did not appear to have any correlation with outcome when observed in *Ras/B‐Raf* wild‐type patients (Figure S5). Similarly, *TP53* alterations are only associated with worse prognosis when they co‐occurred with *Ras/B‐Raf* alterations within the Met group. By contrast, there was no correlation with survival when *TP53* alterations occurred in *Ras/B‐Raf* wild‐type patients within the Met cohort, nor when they occurred in patients from the Adj cohort, regardless of *Ras/B‐Raf* status (Figure S5).

Within the Adj cohort, all three prognostic variables described above (*Ras/B‐Raf* status, *SMAD4* inactivation, and *EGFR* activation) remained significant based on a multivariate analysis using Cox proportional hazards (Figure 3D). Within the Met cohort, extrahepatic disease (*P* = .02) and co‐alteration of *Ras/B‐Raf* and *TP53* (*P* < .001) remained significant factors within the multivariate model, but *Ras/B‐Raf* status was not significant in the absence of *TP53* co‐alteration (Figure 3E). In an independent validation cohort, we note similar trends for survival outcomes, although limited by power, with respect to the mutual exclusivity of *SMAD4* and *Ras/B‐Raf* alterations in the Adj cohort (n = 43), and *TP53* and *Ras/B‐Raf* alterations in the Met cohort (n = 50) (Figure S6).

### 3.4 Molecular stratification of HAI‐treated CLM patients beyond Ras/B‐Raf alterations

**Figure 4:**
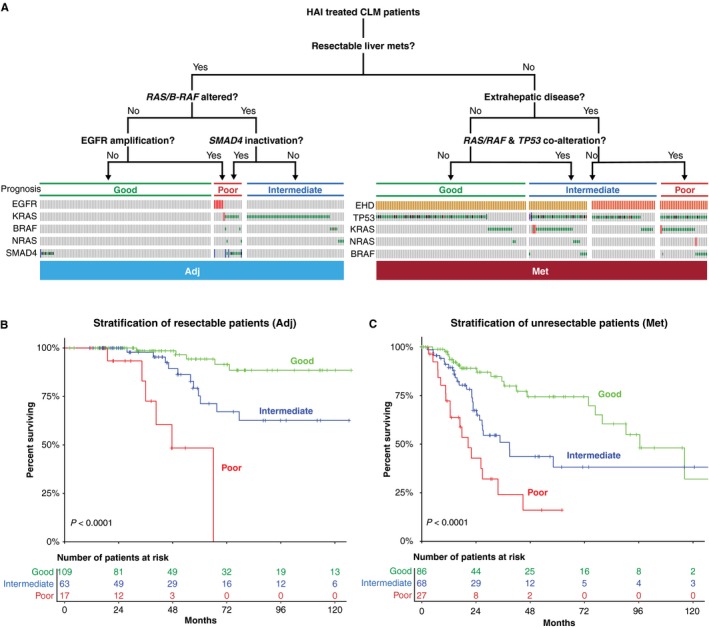
Prognostic stratification of Adj and Met cohorts. (A), Proposed clinico‐pathological and molecular stratification of hepatic arterial infusion‐treated colorectal liver metastasis patients. (B‐C), Kaplan‐Meier curves illustrating differences in overall survival for the stratification proposed in A

CLM patients undergoing HAI have traditionally been divided into resectable and unresectable categories but no distinct molecular characteristics have been described for these groups. As noted above, we observed that these categories relative to the Adj, Met‐EHD(‐), and Met‐EHD(+) classes fall into prognostic groups in terms of overall survival (Figure 1B). Given that we show genomic alterations correlating with OS in patients treated with HAI + systemic chemotherapy, we sought to stratify patients using this information. This molecular stratification consisted of clinical and pathologic features readily available for clinicians (Figure 1B), along with a small number of genomic markers. We then asked if these features could subdivide patients relative to clinical outcomes. We observed differences in prognostic subgroups for the Adjuvant and Met cohorts relative to good, intermediate, and poor prognosis (Figure 4). Taken together, these data suggest that we may be able to refine the prognostic landscape in CLM patients undergoing liver‐directed therapy by including genomic markers, including *Ras/B‐Raf*, *SMAD4*, and *TP53* along with the key clinical characteristics of resectable or unresectable tumors and the presence or absence of extrahepatic disease.

## 4 DISCUSSION

This study represents the largest effort to date at comprehensive molecular characterization, and consequent associations with survival, in CLM patients treated with HAI and systemic chemotherapy. We believe this study is important as we have previously shown that HAI treatment improves outcomes in patients with CLM,^12^ but we also know that not all patients benefit. In this study utilizing NGS in 370 CLM patients undergoing HAI chemotherapy at Memorial Hospital, we implicate key molecular features in addition to *Ras/B‐Raf* in the Adjuvant and Met cohorts. Resectable patients with *Ras/B‐Raf* alteration with co‐occurrent SMAD4 inactivation demonstrated worse outcomes. In addition, for unresectable patients, co‐alteration of *Ras/B‐Raf* and *TP53* was associated with worse outcomes.

Use of HAI in patients with CLM was safe and effective in a randomized controlled trial, demonstrating improved OS in CLM patients who underwent HAI + systemic therapy (68 months) versus systemic therapy alone (55 months).^25^, ^26^ More recently, a retrospective series of 2,368 CLM patients undergoing complete metastasectomy with (n = 785) and without (n = 1583) *perioperative* HAI therapy revealed that patients receiving HAI + systemic chemotherapy had significantly longer OS (67 months) than those treated with systemic therapy alone (44 months).^10^ Despite these encouraging results, molecular markers associated with clinical outcomes, response to therapy, or stratification of prognosis have not been systematically examined in CLM patients receiving HAI. In an analysis examining the impact of *KRAS* mutation *alone* in 169 resected CLM patients treated with HAI and systemic chemotherapy, mutated *KRAS* (codons 12 and 13) was associated with worse outcomes compared with wild‐type *KRAS*.^24^ Previous work using a 33‐gene panel in 193 CLM patients given systemic^4^ treatment followed by curative resection showed that *RAS* mutation was associated with increased recurrence risk due to EHD progression after hepatectomy and shorter OS. In addition, recent work has further implicated *BRAF* alteration as a poor prognostic marker in patients with unresectable metastatic colorectal cancer.^7^ In contrast to these studies, our current study focused on OS in the main study cohort and did not examine patterns of failure after hepatectomy. In the present analysis, an unbiased multiple‐comparison statistical approach—which considers not only *KRAS* and *BRAF* but also 340 + other genes in 370 patients—revealed molecular features in addition to *Ras/B‐Raf* that could be important in a select group of patients in the context of resectable or unresectable disease.

Stratification of clinical outcome by mutational status in CLM patients receiving HAI in the adjuvant or unresectable setting may allow for “molecularly‐guided” selection of patients for available treatment options. In particular, this study is the first to set the stage for more precise patient selection using comprehensive NGS who may benefit from HAI. Since we did not observe any statistically significant differences in terms of frequency of somatic alterations in sequenced primaries vs sequenced metastases, our results suggest that either tumor source could be used indistinctly for this kind of analysis. Based on our data, consideration could be given to aggressive liver‐directed approaches (eg, definitive HAI, HAI as a bridge to resection, two‐stage hepatectomy, etc) for initially unresectable CLM patients with *Ras/B‐Raf* wild‐type tumors. Specifically in the absence of EHD, hepatic disease control appears paramount in this subset of patients where 8‐year OS exceeded 50% (Figure 4). In the presence of EHD, *Ras/B‐Raf* wild‐type patients with initially *unresectable* CLM showed a 5‐year OS approaching 40% following liver‐directed and systemic therapy. Conversely, unresectable patients harboring *Ras/B‐Raf* altered and *TP53* mutant tumors remain a substantial clinical challenge, and these patients did not have the same response as *Ras/B‐Raf* wild‐type patients to liver‐directed therapies in this study.

The association between survival and *SMAD4* loss in CLM patients has been described in patients undergoing resection,^27^ but its importance in patients undergoing liver‐directed chemotherapy (eg, HAI) and systemic therapy has not been previously reported. *SMAD4* is a central mediator of *TGF‐β* signaling whose alteration is observed in 15%‐20% of sporadic CRC cases and correlates with poor overall and disease‐free survival, regardless of tumor stage.^28^ *SMAD4* is an important transcription factor that regulates cell proliferation, differentiation, migration, and apoptosis.^29^ Worse outcome in patients with inactivation of *SMAD4* is perhaps mediated via chemoresistance to 5‐fluorouracil (5‐FU).^30^, ^31^, ^32^ Our data further support this assertion but uncovers an additional layer of complexity in the *Ras/B‐Raf* altered subgroup with concomitant *SMAD4* inactivation, which portends an especially poor prognosis. EGFR amplification in the *Ras/B‐Raf* wild‐type subset was associated with poor prognosis, but given the limited events for this alteration, this finding will need to be further validated in future cohorts. *EGFR* has been identified as a potentially actionable therapeutic target in several cancer types, including metastatic colorectal cancer^33^ and gastroesophageal.^34^

The strength of this report is comprehensive characterization of a fully clinically annotated series of patients with CLM undergoing HAI in a single institution; however, this study has several limitations. First, given the retrospective design, we cannot eliminate biases in determination of resectability or patient selection for liver‐directed approaches and/or surgical resection. Second, our analysis was limited to tumors undergoing sequencing from April 2015 to September 2016, perhaps enriching for a more contemporary cohort in active treatment or long‐term survivors in follow‐up; therefore, we could not ascertain the genomics of the entire CLM population treated with HAI at our institution. Third, with available systemic treatment options and alternative liver‐directed therapies, it remains to be seen if the genetic determinants of outcome in this study are widely applicable to other CLM cohorts.

## 5 CONCLUSIONS

Using comprehensive molecular profiling, alterations in the *Ras/B‐Raf* pathway, together with somatic changes in *SMAD4*, correlate with survival in resectable patients. In unresectable patients, systemic FOLFIRI demonstrates worse OS post‐HAI than 5‐FU/FOLFOX based regimens, and co‐alteration of *Ras/B‐Raf* and *TP53* is a marker of extremely poor outcomes among patients undergoing liver‐directed therapy. These data lay the groundwork for more precise CLM patient selection related to liver‐directed treatment options and provide a template for rational clinical trial design.

## CONFLICT OF INTEREST

One author has received travel support for fellow education from Intuitive Surgical Inc and has served as a clinical advisor to Guardant Health Inc. Another author has received support from this work from Amgen, Inc Another author has received support from Medtronic (honorarium for consultancy with Medtronic), Johnson & Johnson (honorarium delivering a talk), and Intuitive Surgical (honorarium for participating in a webinar by Intuitive Surgical).

## Data Availability

The data that support the findings of this study are available from the corresponding author upon reasonable request.
