# Supplementary material for: Genomic stratification beyond Ras/B‐Raf in colorectal liver metastasis patients treated with hepatic arterial infusion
Source: Cancer Med. 2019 Sep 10;8(15):6538–48. doi: 10.1002/cam4.2415 (PMC6825986; doi:10.1002/cam4.2415)
Supplement: Supplementary file 1 [file CAM4-8-6538-s001.pdf]

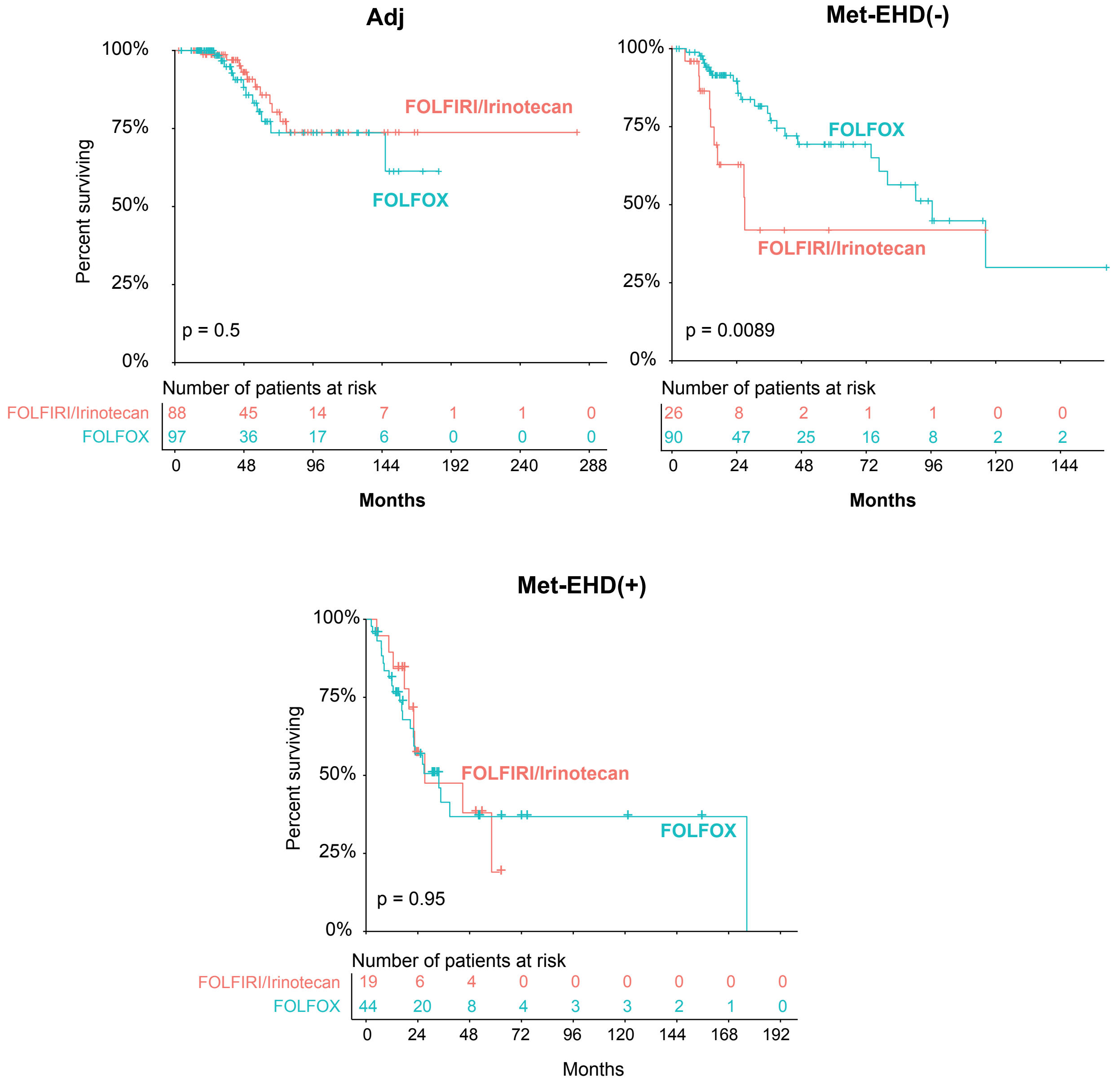

Supplementary Figure 1. Associations between overall survival and chemotherapy regimen, stratified by patient subcohort. FOLFOX = 5-FU based regimens in addition to FOLFOX regimens. FOLFIRI = 5-FU + Leucovorin + Irinotecan.

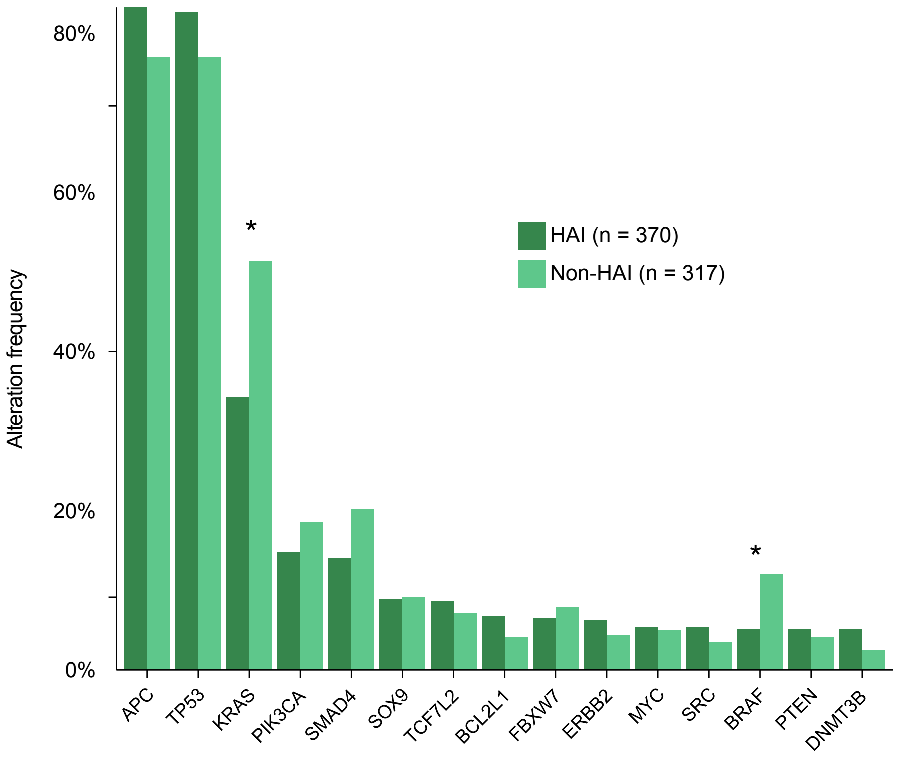

Supplementary Figure 2. Comparison of gene alteration frequencies between the HAI and non-HAI treated patient cohorts. Only KRAS and BRAF exhibited statistically significant differences as noted in the figure by an asterisk*.

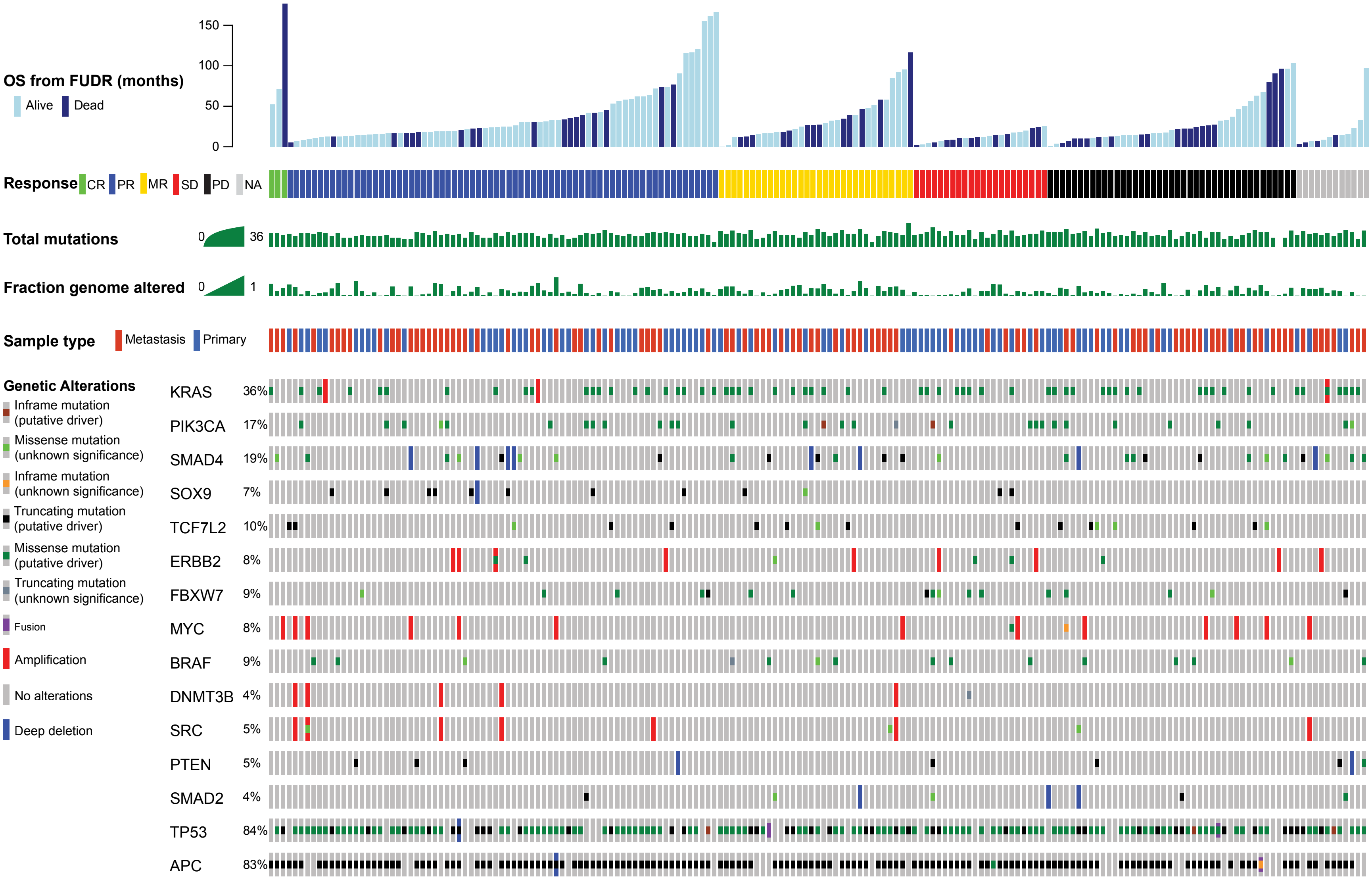

Supplementary Figure 3. Genomic alterations in Met patients stratified by response to treatment.

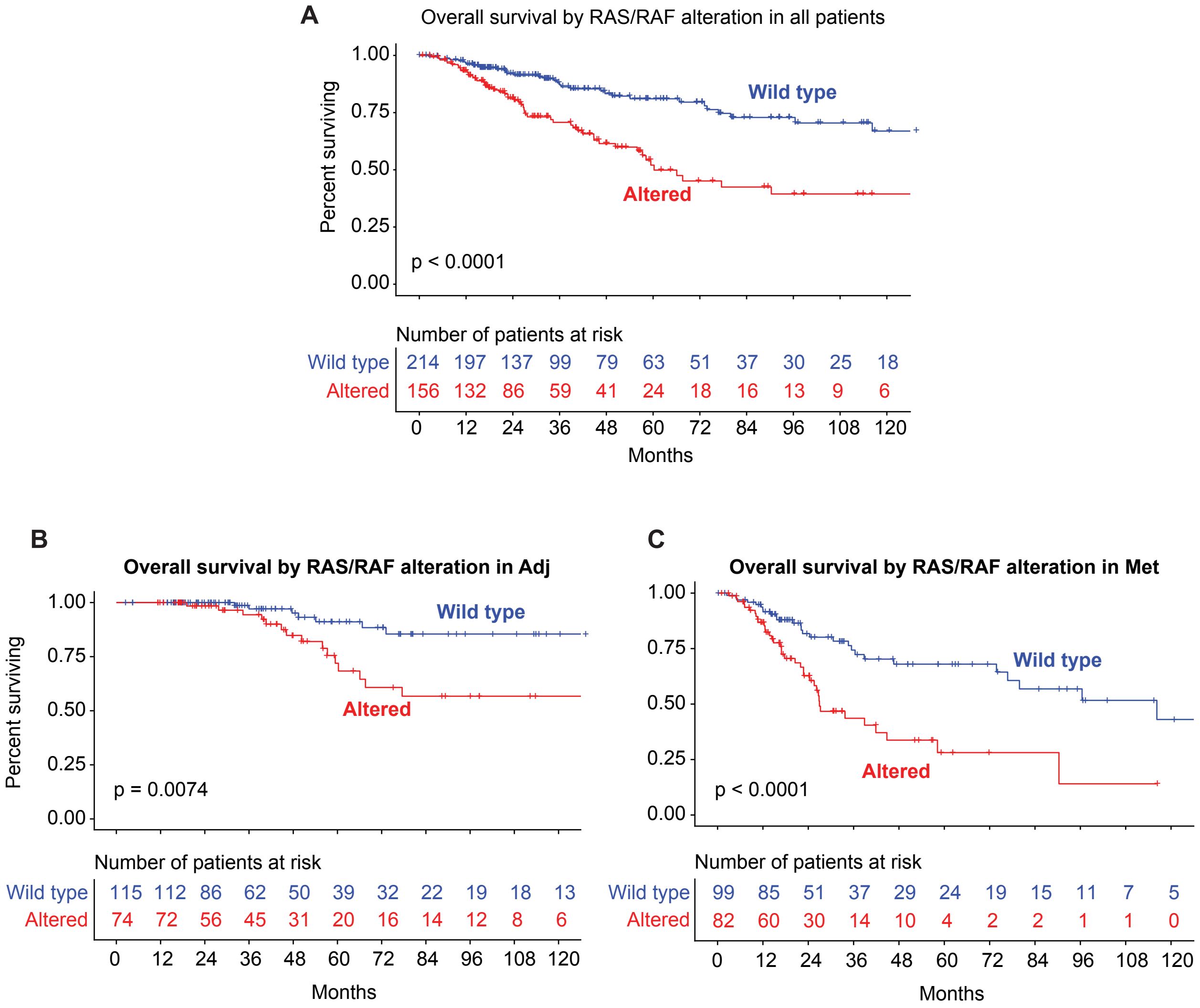

Supplementary Figure 4. Overall survival as a function of Ras/B-Raf status across the Adj, Met-EHD(-) and Met-EHD(+) cohorts.

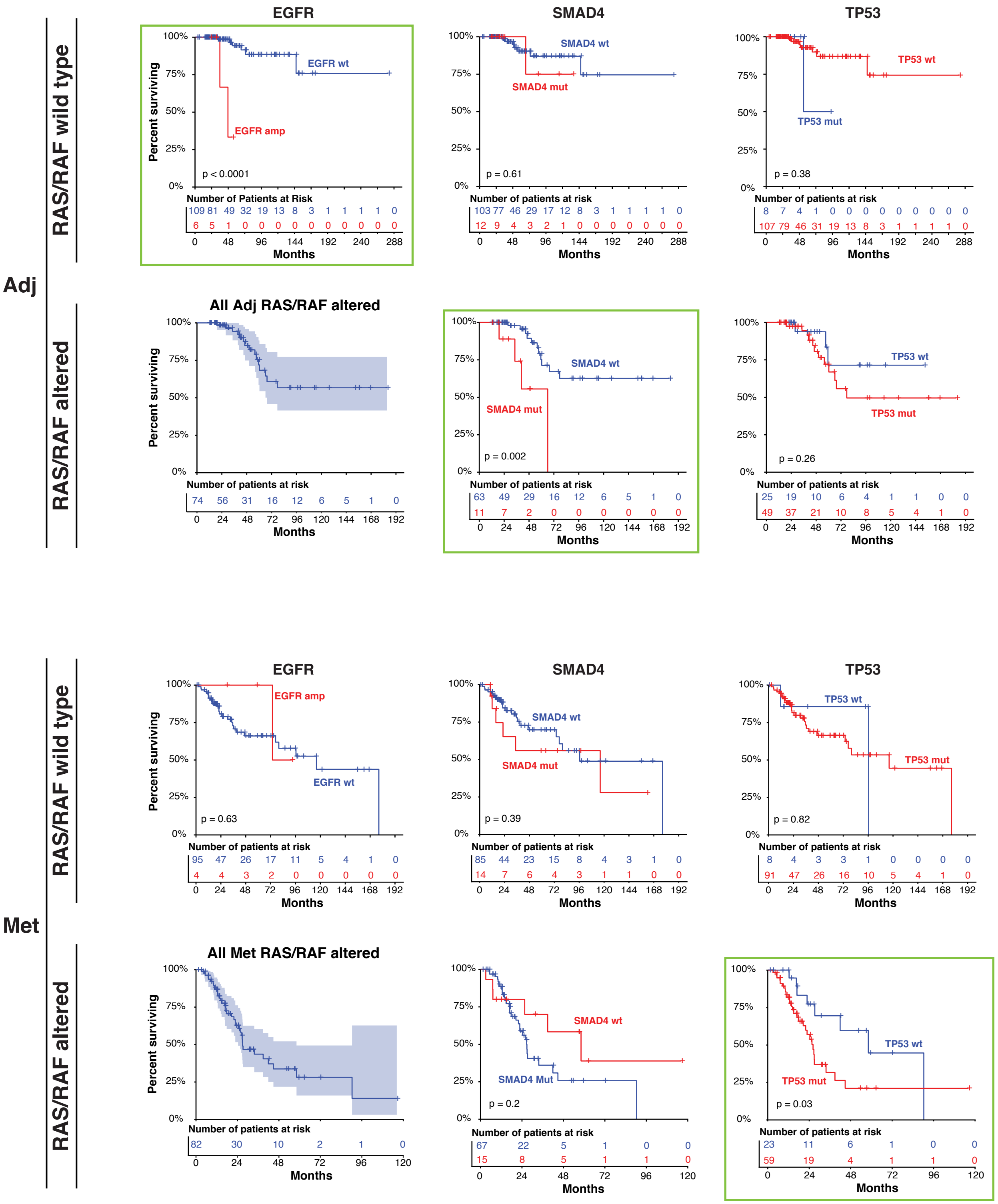

Supplementary Figure 5. Detailed Kaplan-Meier results for EGFR, SMAD4 and TP53 across the Adj, Met-EHD(-) and Met-EHD(+) cohorts.

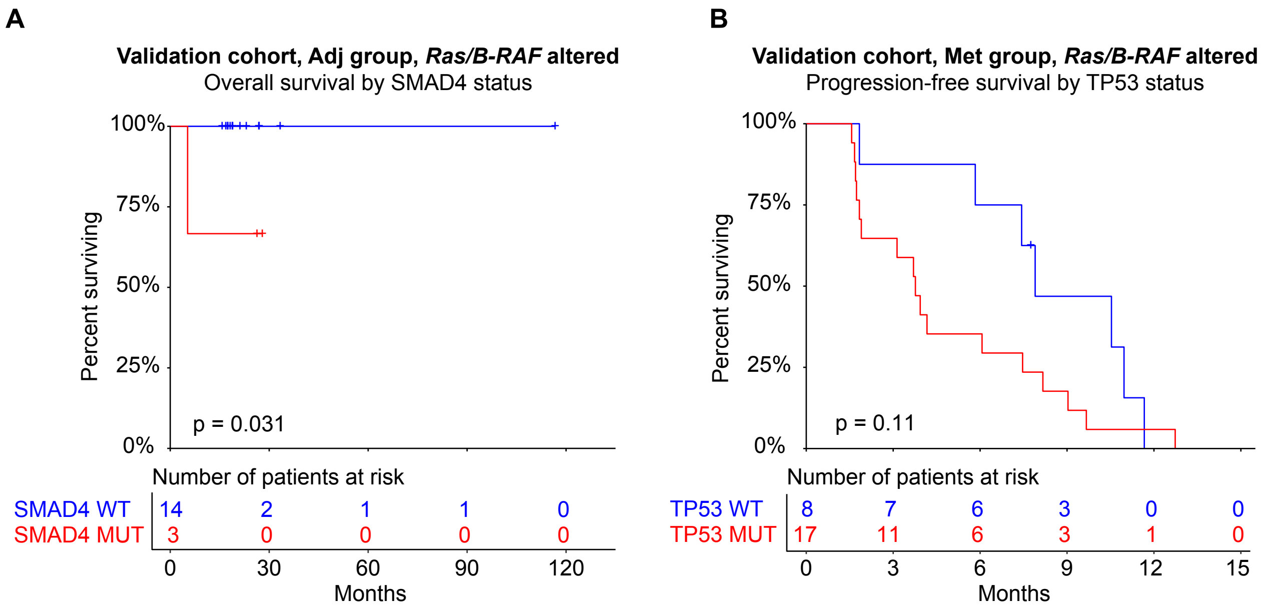

Supplementary Figure 6. Kaplan-Meier results for the validation cohort (n=93). A, Adjuvant (Adj) group patients with Ras/B-Raf alterations stratified by SMAD4 status (WT = wild type; MUT = mutant). B, Metastatic (Met) group patients with Ras/B-Raf alterations stratified by TP53 status. A-B, Log-rank p-values are displayed. Adjuvant and Metastatic groups as defined in Methods.
