# Supplementary material for: Genomic stratification beyond Ras/B‐Raf in colorectal liver metastasis patients treated with hepatic arterial infusion
Source: Cancer Med. 2019 Sep 10;8(15):6538–48. doi: 10.1002/cam4.2415 (PMC6825986; doi:10.1002/cam4.2415)
Supplement: Supplementary file 2 [file CAM4-8-6538-s002.pdf]

Supplementary Table 1a. Adj and Met cohort datasets, part 1

SAMPLE ID	Type	IMPACT PANEL	Race	SEX	AGE FUDR START (yrs)	PRIMARY TUMOR LOCATION	LOCATION SCORE (0=right colon; 1=left colon; 2=rectum)	POSITIVE LN (primary)
P-0000699-T01-IM3	ADJ	341	Asian	M	42	sigmoid colon	1	Y
P-0000736-T01-IM3	ADJ	341	White	F	67	rectosigmoid	2	Y
P-0000737-T01-IM3	ADJ	341	White	F	48	sigmoid colon	1	Y
P-0000738-T01-IM3	ADJ	341	White	M	51	rectosigmoid	2	Y
P-0000742-T01-IM3	ADJ	341	White	M	45	colon	1	Y
P-0000745-T01-IM3	ADJ	341	White	F	73	cecum	0	Y
P-0000762-T01-IM3	ADJ	341	White	M	64	cecum	0	N
P-0000763-T01-IM3	ADJ	341	Black	F	47	sigmoid colon	1	Y
P-0000802-T01-IM3	ADJ	341	White	F	55	sigmoid colon	1	Y
P-0000854-T01-IM3	ADJ	341	Other	M	57	ascending colon	0	Y
P-0000860-T01-IM3	ADJ	341	White	F	50	rectum	2	Y
P-0000868-T01-IM3	ADJ	341	White	F	49	rectosigmoid	2	N
P-0000940-T01-IM3	ADJ	341	White	F	63	hepatic flexure	1	Y
P-0001190-T01-IM3	ADJ	341	White	M	55	descending colon	1	Y
P-0001302-T01-IM3	ADJ	341	White	F	35	sigmoid colon	1	Y
P-0001394-T02-IM5	ADJ	410	White	F	46	colon	0	N
P-0001469-T01-IM3	ADJ	341	White	F	64	sigmoid colon	1	Y
P-0001525-T01-IM3	ADJ	341	Black	M	63	rectosigmoid	2	Y
P-0001531-T01-IM3	ADJ	341	White	M	41	sigmoid colon	1	Y
P-0001534-T01-IM3	ADJ	341	White	M	47	rectosigmoid	2	Y
P-0001984-T01-IM3	ADJ	341	White	F	53	colon	1	N
P-0002035-T01-IM3	ADJ	341	White	F	37	rectosigmoid	2	Y
P-0002268-T01-IM3	ADJ	341	White	F	75	cecum	0	N
P-0002413-T01-IM3	ADJ	341	Asian	M	52	cecum; rectosigmoid	2	N

Supplementary Table 1a continued

SAMPLE ID	Type	IMPACT PANEL	Race	SEX	AGE FUDR START (yrs)	PRIMARY TUMOR LOCATION	LOCATION SCORE (0=right colon; 1=left colon; 2=rectum)	POSITIVE LN (primary)
P-0002702-T01-IM3	ADJ	341	Asian	F	63	large bowel	0	Y
P-0002778-T01-IM3	ADJ	341	White	M	54	cecum	0	Y
P-0002814-T01-IM3	ADJ	341	White	F	57	rectum	2	Y
P-0002835-T01-IM3	ADJ	341	White	M	62	rectosigmoid	2	N
P-0003348-T01-IM5	ADJ	410	White	F	40	rectosigmoid	2	Y
P-0003710-T01-IM5	ADJ	410	White	M	46	rectum	2	N
P-0004754-T01-IM5	ADJ	410	White	M	67	transverse colon and rectum	2	N
P-0004834-T01-IM5	ADJ	410	White	M	62	rectosigmoid	2	N
P-0004960-T01-IM5	ADJ	410	White	M	50	rectum	2	Y
P-0005091-T01-IM5	ADJ	410	White	M	70	rectum	2	N
P-0005237-T01-IM5	ADJ	410	White	F	54	rectosigmoid	2	N
P-0005623-T02-IM5	ADJ	410	Black	F	31	sigmoid colon	1	Y
P-0005715-T01-IM5	ADJ	410	White	M	40	rectum	2	Y
P-0005801-T01-IM5	ADJ	410	Asian	F	50	sigmoid colon	1	Y
P-0006000-T02-IM5	ADJ	410	White	F	41	rectum	2	N
P-0006183-T01-IM5	ADJ	410	Black	M	61	ascending colon	1	N
P-0006213-T01-IM5	ADJ	410	White	M	48	cecum	0	Y
P-0006275-T01-IM5	ADJ	410	White	M	49	rectosigmoid	2	Y
P-0006564-T01-IM5	ADJ	410	White	M	59	sigmoid colon	1	Y
P-0006592-T01-IM5	ADJ	410	White	M	53	sigmoid colon	1	Y
P-0006646-T01-IM5	ADJ	410	White	M	69	sigmoid colon	1	N
P-0006659-T01-IM5	ADJ	410	White	M	49	colon	1	N
P-0006681-T01-IM5	ADJ	410	White	M	59	left colon	1	N
P-0006686-T01-IM5	ADJ	410	White	M	60	cecum	0	N

Supplementary Table 1a continued

SAMPLE ID	Type	IMPACT PANEL	Race	SEX	AGE FUDR START (yrs)	PRIMARY TUMOR LOCATION	LOCATION SCORE (0=right colon; 1=left colon; 2=rectum)	POSITIVE LN (primary)
P-0006701-T01-IM5	ADJ	410	White	M	57	rectum	2	N
P-0006704-T01-IM5	ADJ	410	Black	F	45	cecum and ilocecal valve	0	Y
P-0006711-T01-IM5	ADJ	410	White	F	67	rectum	2	Y
P-0006717-T01-IM5	ADJ	410	White	F	65	ascending colon	0	Y
P-0006735-T01-IM5	ADJ	410	White	M	70	sigmoid colon	1	N
P-0006737-T01-IM5	ADJ	410	White	M	40	rectum	2	Y
P-0006740-T01-IM5	ADJ	410	White	F	66	cecum	0	Y
P-0006744-T01-IM5	ADJ	410	White	M	54	descending colon	1	Y
P-0006759-T01-IM5	ADJ	410	White	M	51	descending colon	0	Y
P-0006761-T01-IM5	ADJ	410	White	F	48	sigmoid colon	1	Y
P-0006763-T01-IM5	ADJ	410	White	M	58	rectum	2	Y
P-0006770-T01-IM5	ADJ	410	Asian	M	45	rectosigmoid	2	Y
P-0006778-T01-IM5	ADJ	410	White	F	68	cecum	0	Y
P-0006816-T01-IM5	ADJ	410	White	F	57	rectum	2	N
P-0006819-T01-IM5	ADJ	410	White	M	62	sigmoid colon	1	N
P-0006821-T01-IM5	ADJ	410	White	M	70	sigmoid colon	1	Y
P-0006822-T01-IM5	ADJ	410	White	M	47	descending colon	1	Y
P-0006888-T01-IM5	ADJ	410	White	F	47	cecum	0	Y
P-0006906-T01-IM5	ADJ	410	White	F	77	ilocecal valve	1	Y
P-0007036-T01-IM5	ADJ	410	White	F	61	rectum	2	Y
P-0007066-T01-IM5	ADJ	410	White	M	42	sigmoid colon	1	Y
P-0007070-T01-IM5	ADJ	410	White	M	73	right colon	0	Y
P-0007079-T01-IM5	ADJ	410	White	M	69	rectum	2	N
P-0007080-T02-IM5	ADJ	410	White	F	57	sigmoid colon	1	Y

Supplementary Table 1a continued

SAMPLE ID	Type	IMPACT PANEL	Race	SEX	AGE FUDR START (yrs)	PRIMARY TUMOR LOCATION	LOCATION SCORE (0=right colon; 1=left colon; 2=rectum)	POSITIVE LN (primary)
P-0007085-T02-IM5	ADJ	410	White	F	74	rectum	2	N
P-0007092-T01-IM5	ADJ	410	White	M	50	rectum	2	Y
P-0007126-T01-IM5	ADJ	410	White	M	48	sigmoid colon	1	Y
P-0007128-T01-IM5	ADJ	410	White	M	71	colon	0	N
P-0007133-T01-IM5	ADJ	410	White	F	60	right colon	0	N
P-0007136-T01-IM5	ADJ	410	White	F	43	transverse colon	0	N
P-0007144-T01-IM5	ADJ	410	Black	M	35	cecum	0	Y
P-0007147-T01-IM5	ADJ	410	Black	F	41	cecum	0	N
P-0007168-T01-IM5	ADJ	410	White	F	68	rectum	2	Y
P-0007175-T01-IM5	ADJ	410	White	F	48	colon	1	Y
P-0007177-T01-IM5	ADJ	410	White	F	66	colon	0	Y
P-0007180-T01-IM5	ADJ	410	White	M	49	sigmoid colon	1	Y
P-0007181-T01-IM5	ADJ	410	White	M	47	rectosigmoid	2	Y
P-0007216-T01-IM5	ADJ	410	White	M	50	cecum	0	Y
P-0007218-T01-IM5	ADJ	410	White	F	56	sigmoid colon	1	Y
P-0007222-T01-IM5	ADJ	410	White	F	72	rectum	2	N
P-0007294-T01-IM5	ADJ	410	White	M	53	rectum	2	N
P-0007305-T01-IM5	ADJ	410	White	M	37	sigmoid colon	1	N
P-0007315-T01-IM5	ADJ	410	White	F	57	rectum	2	Y
P-0007361-T01-IM5	ADJ	410	White	F	77	sigmoid colon	1	N
P-0007368-T01-IM5	ADJ	410	White	M	46	colon	0	N
P-0007373-T01-IM5	ADJ	410	White	M	67	lower sigmoid/rectum	2	N
P-0007380-T01-IM5	ADJ	410	White	M	61	sigmoid colon	1	Y
P-0007085-T02-IM5	ADJ	410	White	F	74	rectum	2	N

Supplementary Table 1a continued

SAMPLE ID	Type	IMPACT PANEL	Race	SEX	AGE FUDR START (yrs)	PRIMARY TUMOR LOCATION	LOCATION SCORE (0=right colon; 1=left colon; 2=rectum)	POSITIVE LN (primary)
P-0007381-T01-IM5	ADJ	410	White	F	44	ascending colon	0	Y
P-0007389-T01-IM5	ADJ	410	Black	F	54	descending colon	1	N
P-0007483-T01-IM5	ADJ	410	White	M	78	ascending colon	1	N
P-0007486-T01-IM5	ADJ	410	Asian	F	50	sigmoid colon	1	Y
P-0007488-T01-IM5	ADJ	410	White	F	44	rectosigmoid	2	Y
P-0007490-T01-IM5	ADJ	410	White	F	55	sigmoid colon	1	Y
P-0007560-T01-IM5	ADJ	410	White	M	63	colon	0	N
P-0007584-T01-IM5	ADJ	410	White	F	67	descending colon	1	Y
P-0007592-T01-IM5	ADJ	410	White	M	54	ascending colon	0	N
P-0007593-T01-IM5	ADJ	410	White	F	72	colon	1	N
P-0007631-T01-IM5	ADJ	410	White	F	43	sigmoid colon	1	Y
P-0007632-T01-IM5	ADJ	410	White	F	69	cecum	0	N
P-0007657-T01-IM5	ADJ	410	White	F	54	sigmoid and rectum	2	N
P-0007664-T01-IM5	ADJ	410	White	M	49	rectum	2	N
P-0007669-T01-IM5	ADJ	410	White	F	43	transverse colon	0	N
P-0007676-T01-IM5	ADJ	410	White	F	55	sigmoid and rectum	2	Y
P-0007686-T01-IM5	ADJ	410	White	M	34	sigmoid colon	1	Y
P-0007691-T01-IM5	ADJ	410	White	F	46	sigmoid colon	1	Y
P-0007740-T01-IM5	ADJ	410	White	F	44	sigmoid colon	1	N
P-0007781-T01-IM5	ADJ	410	White	M	64	sigmoid colon	1	Y
P-0007793-T01-IM5	ADJ	410	White	M	65	colon	1	Y
P-0007860-T01-IM5	ADJ	410	White	M	56	ascending colon	0	Y
P-0007868-T01-IM5	ADJ	410	White	M	56	rectosigmoid	2	N
P-0007869-T01-IM5	ADJ	410	White	F	52	rectum	2	N

Supplementary Table 1a continued

SAMPLE ID	Type	IMPACT PANEL	Race	SEX	AGE FUDR START (yrs)	PRIMARY TUMOR LOCATION	LOCATION SCORE (0=right colon; 1=left colon; 2=rectum)	POSITIVE LN (primary)
P-0007883-T01-IM5	ADJ	410	White	F	44	sigmoid colon	1	Y
P-0007889-T01-IM5	ADJ	410	White	M	50	sigmoid colon	1	Y
P-0007893-T01-IM5	ADJ	410	White	M	68	sigmoid colon	1	Y
P-0007909-T01-IM5	ADJ	410	White	M	62	rectosigmoid	2	Y
P-0007929-T01-IM5	ADJ	410	White	M	60	descending colon	1	N
P-0007931-T01-IM5	ADJ	410	White	M	46	left colon	1	Y
P-0007996-T01-IM5	ADJ	410	White	F	58	rectum	2	N
P-0008068-T01-IM5	ADJ	410	White	F	44	rectum	2	Y
P-0008141-T01-IM5	ADJ	410	White	F	52	rectum	2	N
P-0008167-T01-IM5	ADJ	410	White	M	65	sigmoid colon	1	N
P-0008308-T01-IM5	ADJ	410	White	M	58	rectum	2	N
P-0008357-T01-IM5	ADJ	410	White	M	54	sigmoid colon	1	N
P-0008541-T01-IM5	ADJ	410	White	M	40	rectum	2	Y
P-0012419-T01-IM5	ADJ	410	Asian	M	48	rectal	2	Y
P-0000777-T01-IM3	ADJ	341	White	F	43	sigmoid colon	1	N
P-0001690-T01-IM3	ADJ	341	White	M	45	rectal	2	NA
P-0002032-T01-IM3	ADJ	341	Asian	M	64	rectosigmoid	1	Y
P-0004363-T01-IM5	ADJ	410	Asian	F	35	descending colon	1	Y
P-0004697-T01-IM5	ADJ	410	White	M	66	Splenic flexure	1	Y
P-0004904-T01-IM5	ADJ	410	White	M	68	rectum	2	N
P-0005430-T01-IM5	ADJ	410	White	M	69	transverse colon	0	Y
P-0006764-T01-IM5	ADJ	410	White	F	59	rectal	2	NA
P-0007035-T01-IM5	ADJ	410	White	F	69	sigmoid colon	1	Y
P-0007581-T01-IM5	ADJ	410	White	M	58	sigmoid colon	1	Y

Supplementary Table 1a continued

SAMPLE ID	Type	IMPACT PANEL	Race	SEX	AGE FUDR START (yrs)	PRIMARY TUMOR LOCATION	LOCATION SCORE (0=right colon; 1=left colon; 2=rectum)	POSITIVE LN (primary)
P-0007866-T01-IM5	ADJ	410	White	M	70	rectosigmoid	1	N
P-0007912-T01-IM5	ADJ	410	White	F	45	descending colon	1	Y
P-0008063-T01-IM5	ADJ	410	White	M	41	hepatic flexure	0	N
P-0008259-T01-IM5	ADJ	410	White	F	55	rectal	2	Y
P-0008396-T01-IM5	ADJ	410	White	M	72	descending colon	1	Y
P-0008590-T01-IM5	ADJ	410	White	F	56	cecum	0	Y
P-0008594-T01-IM5	ADJ	410	White	F	55	sigmoid colon	1	Y
P-0008671-T01-IM5	ADJ	410	White	M	52	rectal	2	N
P-0008690-T01-IM5	ADJ	410	Hispanic	M	69	cecum	0	Y
P-0008727-T01-IM5	ADJ	410	White	F	60	rectal	2	Y
P-0009145-T01-IM5	ADJ	410	White	F	57	rectal	2	Y
P-0009341-T01-IM5	ADJ	410	White	F	42	rectosigmoid	1	Y
P-0009433-T01-IM5	ADJ	410	White	F	48	sigmoid colon	1	Y
P-0009707-T01-IM5	ADJ	410	White	M	54	descending colon	1	Y
P-0009741-T01-IM5	ADJ	410	White	M	65	sigmoid colon	1	Y
P-0009798-T01-IM5	ADJ	410	White	M	41	rectal	2	N
P-0009842-T01-IM5	ADJ	410	White	F	63	sigmoid colon	1	Y
P-0009850-T01-IM5	ADJ	410	Hispanic	F	53	rectal	2	Y
P-0009902-T01-IM5	ADJ	410	White	F	48	sigmoid colon	1	Y
P-0009903-T01-IM5	ADJ	410	White	M	71	sigmoid and cecum	1	N
P-0010018-T01-IM5	ADJ	410	White	F	67	sigmoid colon	1	N
P-0010020-T01-IM5	ADJ	410	White	F	60	sigmoid colon	1	Y
P-0010074-T01-IM5	ADJ	410	White	F	50	sigmoid colon	1	Y
P-0010207-T01-IM5	ADJ	410	White	M	42	descending colon	1	Y

Supplementary Table 1a continued

SAMPLE ID	Type	IMPACT PANEL	Race	SEX	AGE FUDR START (yrs)	PRIMARY TUMOR LOCATION	LOCATION SCORE (0=right colon; 1=left colon; 2=rectum)	POSITIVE LN (primary)
P-0010365-T01-IM5	ADJ	410	White	F	33	rectal	2	N
P-0010506-T01-IM5	ADJ	410	White	M	69	rectal	2	Y
P-0010513-T01-IM5	ADJ	410	White	F	53	rectal	2	Y
P-0010665-T01-IM5	ADJ	410	Black	F	53	rectosigmoid	1	N
P-0010734-T01-IM5	ADJ	410	Hispanic	M	56	sigmoid colon	1	Y
P-0010751-T01-IM5	ADJ	410	White	F	65	rectosigmoid	1	Y
P-0010968-T01-IM5	ADJ	410	White	M	66	sigmoid colon	1	N
P-0011158-T01-IM5	ADJ	410	White	M	52	ascending colon	0	N
P-0011255-T01-IM5	ADJ	410	White	M	39	sigmoid colon	1	Y
P-0011484-T01-IM5	ADJ	410	White	F	42	sigmoid colon	1	Y
P-0011533-T01-IM5	ADJ	410	White	F	66	cecum	0	Y
P-0012135-T01-IM5	ADJ	410	White	F	34	rectal	2	Y
P-0012340-T01-IM5	ADJ	410	Black	M	69	Splenic flexure	1	N
P-0012399-T01-IM5	ADJ	410	White	F	63	sigmoid colon	1	Y
P-0012447-T01-IM5	ADJ	410	White	M	49	rectal	2	Y
P-0012566-T01-IM5	ADJ	410	White	M	53	cecum	0	Y
P-0013096-T01-IM5	ADJ	410	White	M	34	rectosigmoid	1	Y
P-0013258-T01-IM5	ADJ	410	White	M	29	cecum	0	Y
P-0013407-T01-IM5	ADJ	410	White	M	54	sigmoid colon	1	Y
P-0013820-T01-IM5	ADJ	410	White	F	62	rectal	2	Y
P-0014252-T01-IM6	ADJ	468	White	M	61	cecum	0	N
P-0001079-T01-IM3	MET_EHD-	341	White	M	73	sigmoid colon	1	Y
P-0001192-T01-IM3	MET_EHD+	341	White	M	55	sigmoid colon	1	N
P-0002251-T01-IM3	MET_EHD-	341	White	M	49	rectal	2	Y

Supplementary Table 1a continued

SAMPLE ID	Type	IMPACT PANEL	Race	SEX	AGE FUDR START (yrs)	PRIMARY TUMOR LOCATION	LOCATION SCORE (0=right colon; 1=left colon; 2=rectum)	POSITIVE LN (primary)
P-0004249-T01-IM5	MET_EHD+	410	White	F	49	Unkown	NA	NA
P-0005124-T01-IM5	MET_EHD-	410	Unknown	F	54	rectal	2	Y
P-0008429-T01-IM5	MET_EHD-	410	White	M	61	sigmoid colon	1	Y
P-0008565-T01-IM5	MET_EHD+	410	White	F	61	rectal	2	N
P-0009058-T01-IM5	MET_EHD-	410	Unknown	F	52	sigmoid colon	1	N
P-0009062-T01-IM5	MET_EHD-	410	White	M	43	rectal	2	Y
P-0009302-T01-IM5	MET_EHD+	410	White	M	45	sigmoid colon	1	NA
P-0009971-T01-IM5	MET_EHD-	410	White	M	45	rectal	2	Y
P-0010062-T01-IM5	MET_EHD-	410	White	F	47	hepatic flexure	0	Y
P-0010238-T01-IM5	MET_EHD-	410	White	F	48	sigmoid colon	1	Y
P-0010363-T01-IM5	MET_EHD-	410	White	F	66	cecum	0	N
P-0010512-T01-IM5	MET_EHD-	410	White	F	52	sigmoid colon	1	Y
P-0010966-T01-IM5	MET_EHD-	410	White	M	48	sigmoid colon	1	N
P-0011144-T01-IM5	MET_EHD+	410	White	F	26	rectosigmoid	1	Y
P-0011182-T01-IM5	MET_EHD-	410	White	F	44	sigmoid colon	1	Y
P-0011184-T01-IM5	MET_EHD-	410	White	M	32	transverse colon	0	NA
P-0011283-T01-IM5	MET_EHD-	410	White	M	46	rectal	2	Y
P-0011288-T01-IM5	MET_EHD-	410	White	F	70	transverse colon	0	Y
P-0012032-T01-IM5	MET_EHD-	410	White	M	46	rectal	2	Y
P-0012383-T01-IM5	MET_EHD-	410	White	F	65	sigmoid colon	1	N
P-0012384-T01-IM5	MET_EHD-	410	White	M	61	rectosigmoid	1	N
P-0012418-T01-IM5	MET_EHD-	410	White	F	59	hepatic flexure	0	NA
P-0012623-T01-IM5	MET_EHD-	410	Hispanic	M	54	descending colon	1	Y
P-0012695-T01-IM5	MET_EHD-	410	White	F	53	rectosigmoid	1	Y

Supplementary Table 1a continued

SAMPLE ID	Type	IMPACT PANEL	Race	SEX	AGE FUDR START (yrs)	PRIMARY TUMOR LOCATION	LOCATION SCORE (0=right colon; 1=left colon; 2=rectum)	POSITIVE LN (primary)
P-0012974-T01-IM5	MET_EHD-	410	White	M	71	sigmoid colon	1	N
P-0013146-T01-IM5	MET_EHD+	410	Hispanic	F	59	ascending colon	0	N
P-0013263-T01-IM5	MET_EHD+	410	White	M	47	sigmoid colon	1	Y
P-0013285-T01-IM5	MET_EHD+	410	White	M	42	rectosigmoid	1	Y
P-0013941-T01-IM5	MET_EHD-	410	White	F	53	cecum	0	Y
P-0013947-T01-IM5	MET_EHD-	410	White	M	55	transverse colon	0	Y
P-0014119-T01-IM5	MET_EHD-	410	Asian	F	47	Splenic flexure	1	Y
P-0014168-T01-IM5	MET_EHD+	410	White	F	46	transverse colon	0	Y
P-0014195-T01-IM6	MET_EHD-	410	White	M	36	cecum	0	Y
P-0014415-T01-IM6	MET_EHD-	410	White	M	50	sigmoid colon	1	N
P-0000695-T01-IM3	MET_EHD+	341	White	M	56	sigmoid colon	1	Y
P-0000714-T01-IM3	MET_EHD-	341	White	F	39	sigmoid colon	1	Y
P-0000721-T01-IM3	MET_EHD+	341	White	F	59	cecum	0	Y
P-0000739-T01-IM3	MET_EHD+	341	White	M	78	sigmoid colon	1	N
P-0000744-T01-IM3	MET_EHD+	341	White	F	62	sigmoid colon	1	N
P-0000754-T01-IM3	MET_EHD+	341	Black	F	47	ascending colon	0	Y
P-0000769-T01-IM3	MET_EHD+	341	White	M	51	sigmoid colon	1	Y
P-0000772-T01-IM3	MET_EHD+	341	White	F	40	rectum	2	Y
P-0000827-T01-IM3	MET_EHD+	341	Black	F	49	rectosigmoid	1	Y
P-0000869-T01-IM3	MET_EHD+	341	White	M	52	rectum	2	N
P-0000885-T01-IM3	MET_EHD-	341	White	F	52	cecum	0	Y
P-0000911-T01-IM3	MET_EHD+	341	White	M	50	rectum	2	Y
P-0000933-T01-IM3	MET_EHD-	341	White	F	41	sigmoid colon	1	N
P-0001054-T01-IM3	MET_EHD+	341	White	F	34	cecum	0	Y

Supplementary Table 1a continued

SAMPLE ID	Type	IMPACT PANEL	Race	SEX	AGE FUDR START (yrs)	PRIMARY TUMOR LOCATION	LOCATION SCORE (0=right colon; 1=left colon; 2=rectum)	POSITIVE LN (primary)
P-0001200-T01-IM3	MET_EHD+	341	White	M	47	rectum	2	NA
P-0001289-T01-IM3	MET_EHD+	341	White	F	62	sigmoid colon	1	Y
P-0001347-T02-IM5	MET_EHD-	410	White	M	45	sigmoid colon	1	N
P-0001391-T01-IM3	MET_EHD-	341	White	M	66	rectum	2	Y
P-0001424-T01-IM3	MET_EHD+	341	White	F	37	ascending colon	0	NA
P-0001499-T01-IM3	MET_EHD+	341	White	M	36	rectum	2	NA
P-0001500-T02-IM5	MET_EHD+	410	White	F	55	rectum	2	Y
P-0001693-T02-IM3	MET_EHD-	341	White	F	52	ascending colon	0	N
P-0001702-T01-IM3	MET_EHD+	341	White	F	48	descending colon	1	Y
P-0001761-T01-IM3	MET_EHD-	341	White	F	60	sigmoid colon	1	NA
P-0001909-T01-IM3	MET_EHD-	341	White	M	56	sigmoid colon	1	Y
P-0001961-T03-IM5	MET_EHD+	410	White	F	68	sigmoid colon	1	Y
P-0001964-T01-IM3	MET_EHD+	341	Black	M	49	cecum	0	Y
P-0002070-T01-IM3	MET_EHD-	341	White	M	61	cecum	0	Y
P-0002095-T01-IM3	MET_EHD+	341	Asian	F	44	ascending colon	0	Y
P-0002438-T01-IM3	MET_EHD-	341	White	M	26	rectosigmoid	1	Y
P-0002442-T01-IM3	MET_EHD-	341	White	M	60	rectosigmoid	1	Y
P-0002721-T01-IM3	MET_EHD-	341	White	M	68	rectum	2	Y
P-0002728-T01-IM3	MET_EHD-	341	White	M	53	sigmoid colon	1	Y
P-0002752-T01-IM3	MET_EHD-	341	White	F	55	sigmoid colon	1	Y
P-0002769-T01-IM3	MET_EHD+	341	White	F	50	sigmoid colon	1	Y
P-0002788-T01-IM3	MET_EHD+	341	White	M	59	ascending colon	0	Y
P-0002804-T01-IM3	MET_EHD-	341	White	M	49	descending colon	1	Y
P-0003474-T01-IM5	MET_EHD+	410	White	F	44	ascending colon	0	Y

Supplementary Table 1a continued

SAMPLE ID	Type	IMPACT PANEL	Race	SEX	AGE FUDR START (yrs)	PRIMARY TUMOR LOCATION	LOCATION SCORE (0=right colon; 1=left colon; 2=rectum)	POSITIVE LN (primary)
P-0003518-T01-IM5	MET_EHD-	410	Asian	F	40	sigmoid colon	1	Y
P-0003789-T01-IM5	MET_EHD-	410	White	M	72	ascending colon	0	N
P-0003999-T01-IM5	MET_EHD+	410	White	M	64	rectosigmoid	1	Y
P-0004018-T01-IM3	MET_EHD+	341	White	M	61	cecum and rectum	2	Y
P-0004140-T02-IM5	MET_EHD-	410	White	M	60	Unkown	0	Y
P-0004251-T01-IM5	MET_EHD+	410	Black	F	51	cecum	0	Y
P-0004322-T01-IM5	MET_EHD-	410	Hispanic	M	43	rectum	2	N
P-0004323-T01-IM5	MET_EHD+	410	White	F	69	sigmoid colon	1	Y
P-0004511-T01-IM5	MET_EHD+	410	White	F	55	left colon	1	N
P-0004626-T01-IM5	MET_EHD-	410	White	M	65	cecum	0	Y
P-0004666-T01-IM5	MET_EHD-	410	White	M	52	sigmoid colon	1	Y
P-0004690-T01-IM5	MET_EHD-	410	White	M	69	rectum	2	Y
P-0004698-T01-IM5	MET_EHD+	410	White	M	38	sigmoid colon	1	Y
P-0004744-T01-IM5	MET_EHD+	410	White	F	58	ascending colon	0	Y
P-0004798-T01-IM5	MET_EHD+	410	White	M	48	Splenic flexure	1	N
P-0004957-T01-IM5	MET_EHD+	410	White	M	62	cecum	0	Y
P-0005003-T01-IM5	MET_EHD-	410	White	M	58	ascending colon	0	Y
P-0005132-T01-IM5	MET_EHD+	410	White	M	53	cecum	0	Y
P-0005164-T01-IM5	MET_EHD-	410	White	F	53	sigmoid colon	1	Y
P-0005343-T01-IM5	MET_EHD-	410	White	M	64	rectal	2	Y
P-0005502-T01-IM5	MET_EHD-	410	White	M	59	descending colon	1	Y
P-0005634-T01-IM5	MET_EHD-	410	Asian	M	32	rectum	2	N
P-0005742-T01-IM5	MET_EHD+	410	White	F	67	cecum	0	Y
P-0005762-T01-IM5	MET_EHD-	410	White	M	39	rectum	2	NA

Supplementary Table 1a continued

SAMPLE ID	Type	IMPACT PANEL	Race	SEX	AGE FUDR START (yrs)	PRIMARY TUMOR LOCATION	LOCATION SCORE (0=right colon; 1=left colon; 2=rectum)	POSITIVE LN (primary)
P-0005858-T01-IM5	MET_EHD+	410	White	F	30	rectal	2	NA
P-0005998-T01-IM5	MET_EHD+	410	White	M	44	sigmoid colon	1	Y
P-0006138-T01-IM5	MET_EHD+	410	White	M	44	cecum	0	N
P-0006271-T01-IM5	MET_EHD+	410	White	M	49	rectosigmoid	1	NA
P-0006329-T01-IM5	MET_EHD-	410	Asian	M	57	rectum	2	Y
P-0006575-T01-IM5	MET_EHD+	410	White	F	50	sigmoid colon	1	Y
P-0006587-T01-IM5	MET_EHD+	410	White	M	51	ascending colon	0	Y
P-0006608-T01-IM5	MET_EHD-	410	White	F	49	sigmoid colon	1	N
P-0006616-T01-IM5	MET_EHD+	410	White	M	51	cecum	0	Y
P-0006657-T01-IM5	MET_EHD-	410	White	M	50	sigmoid colon	1	Y
P-0006658-T01-IM5	MET_EHD-	410	White	M	65	rectosigmoid	1	Y
P-0006683-T01-IM5	MET_EHD-	410	White	M	57	rectum	2	Y
P-0006685-T01-IM5	MET_EHD+	410	White	M	59	rectum	2	N
P-0006693-T01-IM5	MET_EHD-	410	White	M	37	cecum	0	Y
P-0006707-T01-IM5	MET_EHD-	410	White	M	54	sigmoid colon	1	Y
P-0006708-T01-IM5	MET_EHD-	410	White	F	32	sigmoid colon	1	Y
P-0006715-T01-IM5	MET_EHD+	410	White	M	44	descending colon	1	Y
P-0006721-T01-IM5	MET_EHD-	410	White	F	50	rectum	2	N
P-0006729-T01-IM5	MET_EHD-	410	White	F	54	sigmoid colon	1	Y
P-0006743-T01-IM5	MET_EHD+	410	Black	F	32	rectosigmoid	1	Y
P-0006750-T01-IM5	MET_EHD-	410	Arab	M	51	descending colon	1	Y
P-0006794-T01-IM5	MET_EHD-	410	White	F	63	sigmoid colon	1	Y
P-0006835-T01-IM5	MET_EHD-	410	White	F	30	descending colon	1	Y
P-0006853-T01-IM5	MET_EHD-	410	White	F	63	rectum	2	N

Supplementary Table 1a continued

SAMPLE ID	Type	IMPACT PANEL	Race	SEX	AGE FUDR START (yrs)	PRIMARY TUMOR LOCATION	LOCATION SCORE (0=right colon; 1=left colon; 2=rectum)	POSITIVE LN (primary)
P-0006864-T01-IM5	MET_EHD-	410	White	F	52	transverse colon	0	N
P-0006936-T01-IM5	MET_EHD-	410	White	M	53	cecum	0	Y
P-0006978-T01-IM5	MET_EHD-	410	White	F	54	sigmoid colon	1	N
P-0006983-T01-IM5	MET_EHD-	410	White	M	64	rectum	2	N
P-0006999-T02-IM5	MET_EHD+	410	Black	F	59	ascending colon	0	N
P-0007012-T01-IM5	MET_EHD+	410	White	M	60	sigmoid colon	1	NA
P-0007039-T01-IM5	MET_EHD+	410	White	F	45	sigmoid colon	1	Y
P-0007120-T01-IM5	MET_EHD+	410	White	M	58	ascending colon	0	Y
P-0007132-T01-IM5	MET_EHD-	410	Hispanic	M	68	sigmoid colon	1	N
P-0007135-T01-IM5	MET_EHD-	410	Hispanic	M	38	rectum	2	Y
P-0007140-T01-IM5	MET_EHD-	410	Hispanic	M	70	rectosigmoid	1	N
P-0007185-T01-IM5	MET_EHD-	410	White	M	52	sigmoid colon	1	Y
P-0007272-T01-IM5	MET_EHD-	410	White	M	48	cecum	0	Y
P-0007279-T01-IM5	MET_EHD-	410	White	M	39	sigmoid colon	1	Y
P-0007287-T01-IM5	MET_EHD-	410	White	F	64	ascending colon	0	Y
P-0007334-T01-IM5	MET_EHD+	410	White	M	63	rectum	2	NA
P-0007365-T01-IM5	MET_EHD-	410	Asian	F	47	rectum	2	N
P-0007405-T01-IM5	MET_EHD-	410	White	M	53	ascending colon	0	Y
P-0007489-T01-IM5	MET_EHD-	410	White	F	40	left colon	1	Y
P-0007586-T01-IM5	MET_EHD+	410	White	M	46	sigmoid colon	1	Y
P-0007591-T01-IM5	MET_EHD-	410	White	F	67	sigmoid colon	1	Y
P-0007683-T01-IM5	MET_EHD-	410	White	M	55	left colon	0	Y
P-0007739-T01-IM5	MET_EHD-	410	White	F	33	transverse colon	0	Y
P-0007771-T01-IM5	MET_EHD-	410	White	F	50	rectosigmoid	1	N

Supplementary Table 1a continued

SAMPLE ID	Type	IMPACT PANEL	Race	SEX	AGE FUDR START (yrs)	PRIMARY TUMOR LOCATION	LOCATION SCORE (0=right colon; 1=left colon; 2=rectum)	POSITIVE LN (primary)
P-0007887-T01-IM5	MET_EHD-	410	Hispanic	M	59	sigmoid colon	1	N
P-0008022-T02-IM5	MET_EHD+	410	White	M	52	cecum	0	Y
P-0008031-T01-IM5	MET_EHD-	410	White	M	45	rectal	2	NA
P-0008042-T01-IM5	MET_EHD-	410	White	M	43	rectosigmoid	1	N
P-0008075-T01-IM5	MET_EHD-	410	White	F	29	sigmoid colon	1	Y
P-0008079-T01-IM5	MET_EHD+	410	White	M	63	descending colon	1	Y
P-0008083-T01-IM5	MET_EHD-	410	White	M	65	ascending colon	0	Y
P-0008094-T01-IM5	MET_EHD-	410	Hispanic	M	58	sigmoid colon	1	N
P-0008267-T01-IM5	MET_EHD-	410	White	F	40	rectum	2	Y
P-0008483-T01-IM5	MET_EHD+	410	White	F	55	sigmoid colon	1	NA
P-0008534-T01-IM5	MET_EHD-	410	White	M	37	sigmoid colon	1	N
P-0008584-T01-IM5	MET_EHD-	410	White	M	63	sigmoid colon	1	Y
P-0008600-T01-IM5	MET_EHD-	410	White	F	61	sigmoid colon	1	N
P-0009377-T01-IM5	MET_EHD-	410	White	M	66	sigmoid colon	1	N
P-0009488-T01-IM5	MET_EHD-	410	White	F	47	rectal	2	Y
P-0009650-T01-IM5	MET_EHD-	410	Black	M	30	sigmoid colon	1	N
P-0009972-T01-IM5	MET_EHD-	410	Hispanic	M	37	rectosigmoid	1	N
P-0010014-T01-IM5	MET_EHD-	410	White	M	52	sigmoid colon	1	Y
P-0010180-T01-IM5	MET_EHD-	410	White	M	58	sigmoid colon	1	N
P-0010364-T01-IM5	MET_EHD-	410	White	F	52	rectal	2	N
P-0010367-T01-IM5	MET_EHD-	410	White	M	46	rectal	2	N
P-0010406-T01-IM5	MET_EHD-	410	Black	F	47	sigmoid colon	1	Y
P-0011296-T01-IM5	MET_EHD-	410	White	M	53	ascending colon	0	Y
P-0011306-T01-IM5	MET_EHD-	410	White	M	50	rectal	2	Y

Supplementary Table 1a continued

SAMPLE ID	Type	IMPACT PANEL	Race	SEX	AGE FUDR START (yrs)	PRIMARY TUMOR LOCATION	LOCATION SCORE (0=right colon; 1=left colon; 2=rectum)	POSITIVE LN (primary)
P-0011455-T01-IM5	MET_EHD-	410	White	F	58	rectal	2	Y
P-0011520-T01-IM5	MET_EHD-	410	White	F	35	rectal	2	Y
P-0012281-T01-IM5	MET_EHD+	410	White	M	36	sigmoid colon	1	N
P-0012426-T01-IM5	MET_EHD-	410	White	F	51	descending colon	1	Y
P-0012625-T01-IM5	MET_EHD-	410	White	F	65	ascending colon	0	Y
P-0012722-T01-IM5	MET_EHD-	410	White	F	61	descending colon	1	N
P-0012823-T01-IM5	MET_EHD-	410	White	M	49	rectal	2	N
P-0013201-T01-IM5	MET_EHD-	410	White	M	62	ascending colon	0	N
P-0013881-T01-IM5	MET_EHD-	410	White	F	55	cecum	0	Y
P-0013982-T01-IM5	MET_EHD-	410	White	M	42	transverse colon	0	N

Supplementary Table 1b. Adj and Met cohort datasets, part 2

SAMPLE ID	SYNC. DISEASE	ANY CHEMO PRE-PUMP (Y/N)	EXTRAHEP DISEASE PRE-PUMP	EXTRAHEP DISEASE SITE	FIRST SYSTEMIC GIVEN WITH PUMP	LIVER PROG.	VITAL STATUS (1=dead; 0=alive)	LAST FOLLOW UP SINCE FUDR START (months)	LAST FOLLOW UP SINCE DIAGNOSIS (months)
P-0000699-T01-IM3	Y	Y	N	NONE	5FU/LV	Y	0	48.7	53.8
P-0000736-T01-IM3	Y	Y	N	NONE	FOLFIRI/Ptab	N	0	28.1	44.5
P-0000737-T01-IM3	Y	N	N	NONE	FOLFOX	Y	1	67.6	69.8
P-0000738-T01-IM3	Y	Y	N	NONE	FOLFIRI	N	0	33.1	49.7
P-0000742-T01-IM3	Y	Y	N	NONE	FOLFIRI/Ptab	Y	0	40.1	51.4
P-0000745-T01-IM3	N	Y	N	NONE	FOLFIRI	Y	1	40.6	49.7
P-0000762-T01-IM3	Y	Y	N	NONE	FOLFIRI	N	1	60.3	67.4
P-0000763-T01-IM3	N	Y	N	NONE	FOLFIRI	Y	1	34.4	53.8
P-0000802-T01-IM3	Y	Y	N	NONE	CPT	N	0	152.1	160.0
P-0000854-T01-IM3	Y	N	N	NONE	FOLFIRI/Ptab	N	0	39.5	41.3
P-0000860-T01-IM3	Y	Y	N	NONE	FOLFIRI	N	0	114.9	120.6
P-0000868-T01-IM3	Y	Y	N	NONE	FOLFIRI/Bev	Y	0	114.1	118.9
P-0000940-T01-IM3	Y	Y	N	NONE	FOLFOX	Y	1	77.6	82.6
P-0001190-T01-IM3	N	Y	Y	lung	FOLFOX	Y	1	50.3	79.5
P-0001302-T01-IM3	Y	Y	N	NONE	FOLFIRI	Y	1	32.1	42.9
P-0001394-T02-IM5	Y	Y	N	NONE	FOLFOX	N	0	25.3	29.0
P-0001469-T01-IM3	Y	Y	N	NONE	5FU/LV	N	0	29.7	33.6
P-0001525-T01-IM3	N	Y	N	NONE	5FU/LV	Y	0	30.8	47.2
P-0001531-T01-IM3	Y	Y	N	NONE	FOLFIRI	Y	0	24.1	32.8
P-0001534-T01-IM3	Y	Y	N	NONE	FOLFIRI/Ptab	N	0	32.3	36.4
P-0001984-T01-IM3	N	N	N	NONE	FOLFOX	N	0	92.5	138.6
P-0002035-T01-IM3	Y	Y	N	NONE	none	N	0	2.7	8.1
P-0002268-T01-IM3	Y	Y	N	NONE	FOLFIRI	N	0	29.1	33.1
P-0002413-T01-IM3	Y	Y	N	NONE	FOLFOX	N	0	22.0	28.4
P-0002702-T01-IM3	Y	Y	N	NONE	FOLFIRI/Bev	Y	1	66.7	72.0
P-0002778-T01-IM3	Y	Y	N	NONE	FOLFOX	N	0	29.8	34.5

Supplementary Table 1b continued.

SAMPLE ID	SYNC. DISEASE	ANY CHEMO PRE-PUMP (Y/N)	EXTRAHEP DISEASE PRE-PUMP	EXTRAHEP DISEASE SITE	FIRST SYSTEMIC GIVEN WITH PUMP	LIVER PROG.	VITAL STATUS (1=dead; 0=alive)	LAST FOLLOW UP SINCE FUDR START (months)	LAST FOLLOW UP SINCE DIAGNOSIS (months)
P-0002814-T01-IM3	N	Y	N	NONE	FOLFOX	N	0	98.7	130.0
P-0002835-T01-IM3	N	Y	N	NONE	FOLFOX	Y	1	66.1	79.4
P-0003348-T01-IM5	Y	Y	N	NONE	FOLFOX	N	0	90.3	94.9
P-0003710-T01-IM5	N	Y	N	NONE	FOLFIRI	N	0	26.0	66.3
P-0004754-T01-IM5	N	Y	N	NONE	none	N	0	39.7	70.0
P-0004834-T01-IM5	N	Y	N	NONE	none	N	1	73.2	106.4
P-0004960-T01-IM5	Y	Y	Y	lung	5FU/LV	N	0	18.6	25.2
P-0005091-T01-IM5	N	Y	N	NONE	FOLFIRI	Y	0	49.6	76.4
P-0005237-T01-IM5	Y	Y	N	NONE	FOLFOX/Bev	N	0	133.2	137.2
P-0005623-T02-IM5	Y	Y	N	NONE	5FU/LV	N	0	16.0	22.7
P-0005715-T01-IM5	N	Y	N	NONE	FOLFIRI	N	0	22.5	44.7
P-0005801-T01-IM5	Y	Y	N	NONE	none	N	0	21.5	29.6
P-0006000-T02-IM5	Y	Y	N	NONE	FOLFIRI	N	0	16.4	21.5
P-0006183-T01-IM5	N	Y	N	NONE	FOLFIRI	N	0	17.1	25.5
P-0006213-T01-IM5	Y	Y	N	NONE	5FU/LV	Y	1	44.8	51.7
P-0006275-T01-IM5	N	Y	N	NONE	FOLFIRI/Ptab	N	0	20.3	58.0
P-0006564-T01-IM5	Y	Y	N	NONE	FOLFIRI	N	0	17.9	22.8
P-0006592-T01-IM5	Y	N	N	NONE	FOLFIRI	N	0	38.9	41.3
P-0006646-T01-IM5	Y	Y	N	NONE	FOLFIRI	N	0	26.7	33.1
P-0006659-T01-IM5	Y	N	N	NONE	FOLFOX	Y	1	55.9	58.1
P-0006681-T01-IM5	N	N	N	NONE	FOLFOX/Bev	Y	0	120.7	152.7
P-0006686-T01-IM5	N	Y	N	NONE	5FU/LV	N	0	40.3	65.0
P-0006701-T01-IM5	N	Y	N	NONE	none	N	0	18.2	37.3
P-0006704-T01-IM5	N	Y	N	NONE	FOLFIRI	N	0	20.9	36.9
P-0006711-T01-IM5	Y	Y	N	NONE	none	N	0	49.7	55.0
P-0006717-T01-IM5	Y	Y	N	NONE	FOLFOX	N	0	25.3	29.6

Supplementary Table 1b continued.

SAMPLE ID	SYNC. DISEASE	ANY CHEMO PRE-PUMP (Y/N)	EXTRAHEP DISEASE PRE-PUMP	EXTRAHEP DISEASE SITE	FIRST SYSTEMIC GIVEN WITH PUMP	LIVER PROG.	VITAL STATUS (1=dead; 0=alive)	LAST FOLLOW UP SINCE FUDR START (months)	LAST FOLLOW UP SINCE DIAGNOSIS (months)
P-0006735-T01-IM5	N	Y	N	NONE	FOLFIRI/Ptab	Y	0	33.1	47.7
P-0006737-T01-IM5	Y	Y	Y	lung	FOLFOX	Y	0	45.1	48.9
P-0006740-T01-IM5	Y	Y	N	NONE	FOLFOX	N	0	33.6	38.4
P-0006744-T01-IM5	N	Y	N	NONE	FOLFIRI/Ptab	N	0	21.9	57.9
P-0006759-T01-IM5	Y	N	N	NONE	FOLFOX	Y	0	102.4	105.4
P-0006761-T01-IM5	Y	Y	N	NONE	FOLFIRI	Y	0	62.6	80.2
P-0006763-T01-IM5	Y	Y	N	NONE	CPT	N	0	183.4	191.8
P-0006770-T01-IM5	Y	Y	N	NONE	FOLFIRI	N	0	16.3	20.9
P-0006778-T01-IM5	Y	N	Y	lung	FOLFOX	N	0	17.3	19.9
P-0006816-T01-IM5	N	Y	N	NONE	FOLFIRI	N	0	17.5	32.2
P-0006819-T01-IM5	N	Y	N	NONE	5FU/LV	Y	0	80.2	105.4
P-0006821-T01-IM5	Y	Y	N	NONE	5FU/LV	N	0	43.8	58.6
P-0006822-T01-IM5	Y	Y	Y	lung	CPT	N	0	80.7	88.7
P-0006888-T01-IM5	N	Y	N	NONE	5FU/LV	N	0	17.6	29.2
P-0006906-T01-IM5	Y	Y	N	NONE	FOLFOX	N	0	48.2	65.7
P-0007036-T01-IM5	Y	Y	N	NONE	FOLFIRI/Ptab	N	0	51.4	87.3
P-0007066-T01-IM5	Y	N	N	NONE	FOLFIRI	N	0	22.5	24.8
P-0007070-T01-IM5	N	Y	N	NONE	FOLFIRI	N	0	23.5	43.8
P-0007079-T01-IM5	N	Y	N	NONE	FOLFIRI	N	0	26.0	41.1
P-0007080-T02-IM5	N	Y	N	NONE	FOLFOX	N	0	80.4	108.3
P-0007081-T02-IM5	Y	Y	N	NONE	FOLFIRI	N	1	47.7	51.9
P-0007085-T02-IM5	N	N	N	NONE	none	N	0	40.8	67.9
P-0007092-T01-IM5	N	Y	N	NONE	FOLFIRI/Ptab	N	0	25.2	39.5
P-0007126-T01-IM5	N	Y	N	NONE	FOLFIRI/Ptab	Y	0	58.5	73.4
P-0007128-T01-IM5	Y	Y	N	NONE	FOLFOX	N	0	114.0	117.7
P-0007133-T01-IM5	Y	Y	N	NONE	CPT	N	0	89.5	101.3

Supplementary Table 1b continued.

SAMPLE ID	SYNC. DISEASE	ANY CHEMO PRE-PUMP (Y/N)	EXTRAHEP DISEASE PRE-PUMP	EXTRAHEP DISEASE SITE	FIRST SYSTEMIC GIVEN WITH PUMP	LIVER PROG.	VITAL STATUS (1=dead; 0=alive)	LAST FOLLOW UP SINCE FUDR START (months)	LAST FOLLOW UP SINCE DIAGNOSIS (months)
P-0007136-T01-IM5	Y	Y	N	NONE	FOLFOX	Y	0	112.5	122.1
P-0007144-T01-IM5	Y	Y	N	NONE	FOLFIRI	N	0	24.4	29.3
P-0007147-T01-IM5	Y	Y	N	NONE	FOLFIRI	N	0	56.6	63.3
P-0007168-T01-IM5	Y	Y	N	NONE	FOLFIRI/Ptab	N	0	27.1	32.2
P-0007175-T01-IM5	Y	Y	N	NONE	5FU/LV	N	0	71.1	78.0
P-0007177-T01-IM5	Y	N	N	NONE	FOLFIRI	Y	0	55.3	69.7
P-0007180-T01-IM5	Y	Y	N	NONE	FOLFOX	N	0	47.6	51.6
P-0007181-T01-IM5	Y	Y	N	NONE	CPT	N	0	149.1	157.2
P-0007216-T01-IM5	Y	N	N	NONE	FOLFOX	Y	0	75.3	78.1
P-0007218-T01-IM5	N	Y	N	NONE	5FU/LV	Y	0	279.6	294.3
P-0007222-T01-IM5	N	Y	N	NONE	5FU/LV	N	0	166.6	193.0
P-0007294-T01-IM5	Y	Y	N	NONE	FOLFOX	Y	0	35.0	38.8
P-0007305-T01-IM5	Y	Y	N	NONE	FOLFIRI/Ptab	N	0	18.3	26.0
P-0007315-T01-IM5	N	Y	N	NONE	5FU/LV	N	0	22.1	64.7
P-0007361-T01-IM5	Y	Y	N	NONE	FOLFIRI	N	0	98.7	104.4
P-0007368-T01-IM5	Y	Y	N	NONE	FOLFIRI	Y	1	57.3	70.5
P-0007373-T01-IM5	N	Y	N	NONE	FOLFIRI	N	0	34.3	63.0
P-0007380-T01-IM5	Y	Y	N	NONE	CPT	N	0	38.3	70.7
P-0007381-T01-IM5	N	Y	N	NONE	FOLFIRI	N	0	29.8	44.2
P-0007389-T01-IM5	N	Y	N	NONE	5FU/LV	Y	0	50.9	79.7
P-0007483-T01-IM5	Y	Y	N	NONE	CPT	N	0	58.8	85.8
P-0007486-T01-IM5	Y	N	N	NONE	FOLFIRI	Y	1	49.4	51.2
P-0007488-T01-IM5	Y	Y	N	NONE	FOLFOX	N	0	50.4	55.4
P-0007490-T01-IM5	Y	N	N	NONE	FOLFIRI	N	0	35.8	37.9
P-0007560-T01-IM5	Y	Y	N	NONE	FOLFIRI	Y	1	27.7	36.5
P-0007584-T01-IM5	Y	Y	N	NONE	FOLFIRI/Bev	N	0	96.3	112.7

Supplementary Table 1b continued.

SAMPLE ID	SYNC. DISEASE	ANY CHEMO PRE-PUMP (Y/N)	EXTRAHEP DISEASE PRE-PUMP	EXTRAHEP DISEASE SITE	FIRST SYSTEMIC GIVEN WITH PUMP	LIVER PROG.	VITAL STATUS (1=dead; 0=alive)	LAST FOLLOW UP SINCE FUDR START (months)	LAST FOLLOW UP SINCE DIAGNOSIS (months)
P-0007592-T01-IM5	N	Y	N	NONE	FOLFIRI	N	0	38.9	60.9
P-0007593-T01-IM5	Y	Y	N	NONE	CPT	N	0	155.6	163.6
P-0007631-T01-IM5	Y	N	N	NONE	FOLFOX	Y	0	77.4	79.2
P-0007632-T01-IM5	Y	Y	N	NONE	FOLFIRI	Y	0	59.4	63.8
P-0007657-T01-IM5	N	Y	N	NONE	FOLFIRI	N	0	43.7	64.8
P-0007664-T01-IM5	Y	Y	N	NONE	FOLFOX	N	0	57.3	61.6
P-0007669-T01-IM5	N	N	N	NONE	FOLFOX	N	0	77.2	128.5
P-0007676-T01-IM5	Y	N	N	NONE	FOLFOX	Y	0	155.8	159.0
P-0007686-T01-IM5	N	Y	N	NONE	5FU/LV	N	0	18.0	39.1
P-0007691-T01-IM5	N	Y	N	NONE	FOLFIRI	N	0	21.9	38.9
P-0007740-T01-IM5	N	Y	N	NONE	FOLFOX	N	0	63.5	105.1
P-0007781-T01-IM5	N	Y	N	NONE	FOLFOX	N	0	168.8	211.5
P-0007793-T01-IM5	Y	Y	N	NONE	FOLFIRI	N	0	42.1	46.8
P-0007860-T01-IM5	Y	Y	N	NONE	FOLFIRI	Y	1	54.1	59.9
P-0007868-T01-IM5	N	N	N	NONE	FOLFOX	N	0	113.5	131.3
P-0007869-T01-IM5	N	Y	N	NONE	FOLFOX	N	0	43.4	79.0
P-0007883-T01-IM5	N	Y	N	NONE	FOLFOX	N	0	72.7	93.6
P-0007889-T01-IM5	Y	Y	N	NONE	FOLFIRI	N	0	127.6	136.7
P-0007893-T01-IM5	Y	Y	N	NONE	FOLFIRI	N	0	72.5	123.6
P-0007909-T01-IM5	Y	Y	N	NONE	FOLFOX	N	0	148.1	155.6
P-0007929-T01-IM5	Y	N	N	NONE	FOLFOX	N	0	42.3	44.5
P-0007931-T01-IM5	N	Y	N	NONE	FOLFIRI	N	0	29.4	69.8
P-0007996-T01-IM5	Y	Y	N	NONE	FOLFIRI	N	0	25.7	52.7
P-0008068-T01-IM5	Y	Y	N	NONE	FOLFIRI	N	0	64.5	83.3
P-0008141-T01-IM5	N	Y	N	NONE	FOLFOX	N	0	17.0	30.1
P-0008167-T01-IM5	N	N	N	NONE	CPT	N	1	146.3	177.5

Supplementary Table 1b continued.

SAMPLE ID	SYNC. DISEASE	ANY CHEMO PRE-PUMP (Y/N)	EXTRAHEP DISEASE PRE-PUMP	EXTRAHEP DISEASE SITE	FIRST SYSTEMIC GIVEN WITH PUMP	LIVER PROG.	VITAL STATUS (1=dead; 0=alive)	LAST FOLLOW UP SINCE FUDR START (months)	LAST FOLLOW UP SINCE DIAGNOSIS (months)
P-0008308-T01-IM5	Y	Y	N	NONE	5FU/LV	Y	1	36.0	53.8
P-0008357-T01-IM5	N	Y	N	NONE	5FU/LV	N	0	54.0	98.8
P-0008541-T01-IM5	N	Y	N	NONE	FOLFIRI/Ptab	N	0	43.3	60.9
P-0012419-T01-IM5	Y	Y	N	NONE	FOLFIRI	N	0	4.6	12.0
P-0000777-T01-IM3	N	N	N	NONE	FOLFOX	N	0	94.9	131.1
P-0001690-T01-IM3	Y	Y	N	NONE	5-FU + LEUCOVORIN	N	0	26.0	35.2
P-0002032-T01-IM3	Y	Y	Y	lung	Capectabine	Y	0	32.5	49.8
P-0004363-T01-IM5	Y	Y	N	NONE	FOLFOX	Y	1	19.1	24.7
P-0004697-T01-IM5	Y	Y	N	NONE	FOLFOX	Y	0	27.3	35.2
P-0004904-T01-IM5	N	Y	Y	LN	CPT	N	0	23.4	58.5
P-0005430-T01-IM5	N	Y	N	NONE	IRINOTECAN	Y	0	18.0	40.7
P-0006764-T01-IM5	Y	Y	Y	lung + pelvis	FOLFIRI	N	0	47.9	58.5
P-0007035-T01-IM5	Y	Y	Y	lung	5-FU + LEUCOVORIN	N	0	40.1	63.1
P-0007581-T01-IM5	Y	Y	Y	lung	5-FU + LEUCOVORIN	Y	1	46.2	52.5
P-0007866-T01-IM5	Y	Y	N	NONE	FOLFIRI	Y	0	31.1	40.7
P-0007912-T01-IM5	Y	Y	Y	Ovary, peritoneum	FOLFOX	Y	0	60.8	77.8
P-0008063-T01-IM5	Y	Y	N	NONE	5-FU + LEUCOVORIN	N	0	56.3	70.6
P-0008259-T01-IM5	Y	Y	N	NONE	FOLFOX	N	0	15.8	21.6
P-0008396-T01-IM5	Y	Y	N	NONE	FOLFOX	N	0	145.6	157.8
P-0008590-T01-IM5	Y	Y	Y	lung	FOLFOX	N	1	59.4	65.5
P-0008594-T01-IM5	Y	Y	N	NONE	5-FU	Y	0	19.7	33.9
P-0008671-T01-IM5	N	Y	N	NONE	FOLFOX	N	0	88.6	102.5

Supplementary Table 1b continued.

SAMPLE ID	SYNC. DISEASE	ANY CHEMO PRE-PUMP (Y/N)	EXTRAHEP DISEASE PRE-PUMP	EXTRAHEP DISEASE SITE	FIRST SYSTEMIC GIVEN WITH PUMP	LIVER PROG.	VITAL STATUS (1=dead; 0=alive)	LAST FOLLOW UP SINCE FUDR START (months)	LAST FOLLOW UP SINCE DIAGNOSIS (months)
P-0008690-T01-IM5	N	Y	N	NONE	FOLFIRI	Y	0	17.9	32.7
P-0008727-T01-IM5	Y	Y	N	NONE	FOLFIRI	N	0	126.6	133.1
P-0009145-T01-IM5	N	Y	N	NONE	5-FU + LEUCOVORIN	N	0	16.0	32.5
P-0009341-T01-IM5	N	Y	N	NONE	PTAB	Y	0	17.7	32.3
P-0009433-T01-IM5	Y	Y	N	NONE	FOLFIRI	Y	0	11.5	27.4
P-0009707-T01-IM5	Y	Y	N	NONE	FOLFIRI + PTAB	Y	0	32.9	58.6
P-0009741-T01-IM5	N	Y	N	NONE	5-FU + LEUCOVORIN	N	0	83.4	104.6
P-0009798-T01-IM5	N	Y	Y	lung	5-FU + LEUCOVORIN	N	0	17.0	37.1
P-0009842-T01-IM5	Y	Y	N	NONE	NONE	Y	0	16.3	45.3
P-0009850-T01-IM5	N	Y	N	NONE	FOLFIRI	Y	0	30.5	47.6
P-0009902-T01-IM5	Y	Y	N	NONE	5-FU + LEUCOVORIN	Y	0	45.6	66.1
P-0009903-T01-IM5	Y	Y	N	NONE	FOLFOX	N	0	153.5	180.7
P-0010018-T01-IM5	N	Y	N	NONE	FOLFIRI	N	0	63.5	77.5
P-0010020-T01-IM5	Y	N	N	NONE	IRINOTECAN	N	0	172.4	230.8
P-0010074-T01-IM5	Y	Y	N	NONE	FOLFIRI	N	0	135.0	145.5
P-0010207-T01-IM5	N	Y	N	NONE	FOLFIRI + PTAB	N	0	15.9	24.3
P-0010365-T01-IM5	Y	Y	N	NONE	5FU + IRINOTECAN	N	0	11.5	17.1
P-0010506-T01-IM5	N	Y	N	NONE	FOLFIRI	N	0	15.6	45.0
P-0010513-T01-IM5	N	Y	Y	lung	IRINOTECAN	N	0	66.3	83.0
P-0010665-T01-IM5	N	Y	N	NONE	FOLFIRI	N	0	134.7	192.9
P-0010734-T01-IM5	Y	Y	N	NONE	IRINOTECAN	Y	0	25.2	35.1

Supplementary Table 1b continued.

SAMPLE ID	SYNC. DISEASE	ANY CHEMO PRE-PUMP (Y/N)	EXTRAHEP DISEASE PRE-PUMP	EXTRAHEP DISEASE SITE	FIRST SYSTEMIC GIVEN WITH PUMP	LIVER PROG.	VITAL STATUS (1=dead; 0=alive)	LAST FOLLOW UP SINCE FUDR START (months)	LAST FOLLOW UP SINCE DIAGNOSIS (months)
P-0010751-T01-IM5	N	Y	N	NONE	5-FU	N	0	76.8	101.7
P-0010968-T01-IM5	Y	Y	N	NONE	IRINOTECAN	N	0	15.1	28.0
P-0011158-T01-IM5	N	Y	N	NONE	FOLFIRI	Y	1	39.5	71.3
P-0011255-T01-IM5	Y	Y	N	NONE	FOLFIRI	N	0	108.9	112.6
P-0011484-T01-IM5	Y	Y	N	NONE	FOLFIRI + BEVACIZUMAB	N	0	116.9	125.5
P-0011533-T01-IM5	Y	Y	N	NONE	FOLFIRI	Y	0	96.3	118.3
P-0012135-T01-IM5	Y	Y	N	NONE	FOLFIRI	Y	0	18.9	27.3
P-0012340-T01-IM5	N	Y	N	NONE	FOLFOX	N	0	14.0	25.1
P-0012399-T01-IM5	N	Y	N	NONE	NONE	N	0	14.1	70.9
P-0012447-T01-IM5	N	Y	N	NONE	NONE	N	0	12.9	36.7
P-0012566-T01-IM5	N	Y	N	NONE	IRINOTECAN	Y	0	46.1	61.9
P-0013096-T01-IM5	Y	Y	N	NONE	FOLFOX	Y	0	40.9	46.2
P-0013258-T01-IM5	Y	N	N	NONE	FOLFOX	N	0	13.1	14.8
P-0013407-T01-IM5	N	Y	N	NONE	NONE	N	0	12.0	16.4
P-0013820-T01-IM5	Y	Y	N	NONE	FOLFIRI + PTAB	N	0	4.7	13.3
P-0014252-T01-IM6	N	Y	N	NONE	5-FU	Y	0	52.0	74.6
P-0001079-T01-IM3	Y	Y	N	NONE	IRINOTECAN	Y	1	30.6	36.6
P-0001192-T01-IM3	N	Y	Y	lung	FOLFIRI	N	0	73.9	163.7
P-0002251-T01-IM3	Y	Y	N	NONE	FOLFIRI	Y	0	36.8	44.8
P-0004249-T01-IM5	Y	Y	Y	Ovary	FOLFIRI	Y	1	7.2	19.4
P-0005124-T01-IM5	Y	Y	N	NONE	5-FU + LEUCOVORIN	Y	1	10.2	17.1
P-0008429-T01-IM5	Y	Y	N	NONE	5-FU	Y	1	16.9	19.2
P-0008565-T01-IM5	Y	Y	Y	lung	5-FU + LEUCOVORIN	Y	0	14.3	41.9

Supplementary Table 1b continued.

SAMPLE ID	SYNC. DISEASE	ANY CHEMO PRE-PUMP (Y/N)	EXTRAHEP DISEASE PRE-PUMP	EXTRAHEP DISEASE SITE	FIRST SYSTEMIC GIVEN WITH PUMP	LIVER PROG.	VITAL STATUS (1=dead; 0=alive)	LAST FOLLOW UP SINCE FUDR START (months)	LAST FOLLOW UP SINCE DIAGNOSIS (months)
P-0009058-T01-IM5	Y	Y	N	NONE	FOLFIRI	Y	0	32.8	45.9
P-0009062-T01-IM5	Y	Y	N	NONE	IRINOTECAN	Y	0	14.4	27.6
P-0009302-T01-IM5	Y	Y	Y	Peritoneum	FOLFIRI	Y	0	13.6	22.0
P-0009971-T01-IM5	Y	Y	N	NONE	IRINOTECAN	Y	1	12.0	27.1
P-0010062-T01-IM5	Y	Y	N	NONE	5-FU + LEUCOVORIN	N	1	10.0	27.6
P-0010238-T01-IM5	Y	Y	N	NONE	FOLFIRI	Y	0	67.3	72.1
P-0010363-T01-IM5	Y	Y	N	NONE	FOLFIRI	N	0	16.7	27.8
P-0010512-T01-IM5	Y	Y	N	NONE	NONE YET	N	0	0.1	17.9
P-0010966-T01-IM5	Y	Y	N	NONE	FOLFIRI + BEVACIZUMAB	Y	0	13.1	42.3
P-0011144-T01-IM5	Y	Y	Y	Ovary	FOLFOX	Y	1	10.6	15.1
P-0011182-T01-IM5	Y	Y	N	NONE	FOLFOX + PTAB	Y	1	15.6	19.8
P-0011184-T01-IM5	Y	Y	N	NONE	5FU	N	0	8.2	19.7
P-0011283-T01-IM5	Y	Y	N	NONE	FOLFIRI	Y	0	18.5	62.6
P-0011288-T01-IM5	Y	Y	N	NONE	5-FU + LEUCOVORIN	Y	0	25.5	34.4
P-0012032-T01-IM5	N	Y	N	NONE	FOLFIRI	N	0	16.1	27.0
P-0012383-T01-IM5	N	Y	N	NONE	FOLFOX	Y	1	4.8	43.4
P-0012384-T01-IM5	Y	Y	N	NONE	FOLFIRI	N	0	15.0	29.0
P-0012418-T01-IM5	Y	Y	N	NONE	IRINOTECAN	Y	0	12.0	18.5
P-0012623-T01-IM5	Y	Y	N	NONE	IRINOTECAN + OXALIPLATIN	N	0	13.7	17.9
P-0012695-T01-IM5	Y	Y	N	NONE	IRINOTECAN + PTAB	N	0	14.0	31.2
P-0012974-T01-IM5	Y	Y	N	NONE	FOLFIRI	Y	0	10.7	15.8

Supplementary Table 1b continued.

SAMPLE ID	SYNC. DISEASE	ANY CHEMO PRE-PUMP (Y/N)	EXTRAHEP DISEASE PRE-PUMP	EXTRAHEP DISEASE SITE	FIRST SYSTEMIC GIVEN WITH PUMP	LIVER PROG.	VITAL STATUS (1=dead; 0=alive)	LAST FOLLOW UP SINCE FUDR START (months)	LAST FOLLOW UP SINCE DIAGNOSIS (months)
P-0013146-T01-IM5	Y	Y	Y	lung + ovaries	IRINOTECAN	Y	0	3.7	15.7
P-0013263-T01-IM5	Y	Y	Y	Umbilicus	FOLFIRI	Y	0	4.8	16.3
P-0013285-T01-IM5	N	Y	Y	Umbilicus	FOLFIRI	Y	0	13.2	37.3
P-0013941-T01-IM5	Y	Y	N	NONE	IRINOTECAN + PTAB	Y	0	6.5	19.7
P-0013947-T01-IM5	Y	Y	N	NONE	FOLFOX	Y	0	7.1	12.5
P-0014119-T01-IM5	Y	Y	N	NONE	FOLFIRI	N	0	11.6	14.1
P-0014168-T01-IM5	Y	Y	Y	lung	FOLFIRI	N	0	11.3	15.3
P-0014195-T01-IM6	Y	Y	N	NONE	FOLFIRI	N	0	2.7	8.0
P-0014415-T01-IM6	Y	Y	N	NONE	IRINOTECAN + OXALIPLATIN	N	0	18.9	23.3
P-0000695-T01-IM3	Y	Y	Y	soft tissue, LN, adrenal, lung	FOLFIRI	N	1	2.4	28.8
P-0000714-T01-IM3	Y	Y	N	NONE	FOLFIRI	Y	1	10.2	13.7
P-0000721-T01-IM3	Y	N	Y	Peritoneum	CPT/oxali	Y	1	22.8	25.3
P-0000739-T01-IM3	Y	Y	Y	LN, lung	CPT	Y	0	52.0	70.1
P-0000744-T01-IM3	Y	Y	Y	LN	5FU/LV	Y	1	58.2	64.4
P-0000754-T01-IM3	Y	Y	Y	lung	CPT	Y	1	12.6	23.0
P-0000769-T01-IM3	Y	N	Y	Lung, LN	CPT/oxali	Y	0	120.7	122.3
P-0000772-T01-IM3	Y	Y	Y	lung	CPT	Y	1	38.8	61.1
P-0000827-T01-IM3	Y	Y	Y	lung	5-FU + LEUCOVORIN	Y	1	22.1	26.1
P-0000869-T01-IM3	Y	Y	Y	LN	FOLFIRI	N	0	51.5	58.2
P-0000885-T01-IM3	Y	Y	N	NONE	FOLFOX	N	0	41.7	45.2
P-0000911-T01-IM3	Y	Y	Y	LN	FOLFOX	Y	1	19.9	30.6
P-0000933-T01-IM3	Y	Y	N	NONE	FOLFIRI	Y	1	22.8	30.8

Supplementary Table 1b continued.

SAMPLE ID	SYNC. DISEASE	ANY CHEMO PRE-PUMP (Y/N)	EXTRAHEP DISEASE PRE-PUMP	EXTRAHEP DISEASE SITE	FIRST SYSTEMIC GIVEN WITH PUMP	LIVER PROG.	VITAL STATUS (1=dead; 0=alive)	LAST FOLLOW UP SINCE FUDR START (months)	LAST FOLLOW UP SINCE DIAGNOSIS (months)
P-0001054-T01-IM3	Y	Y	Y	Peritoneum, LN	FOLFIRI	N	0	31.1	36.3
P-0001200-T01-IM3	Y	Y	Y	LN	FOLFIRI	N	0	32.2	41.6
P-0001289-T01-IM3	Y	Y	Y	Peritoneum	FOLFOX	Y	0	53.1	59.3
P-0001347-T02-IM5	Y	Y	N	NONE	FOLFIRI	Y	0	32.1	41.7
P-0001391-T01-IM3	Y	Y	N	NONE	FOLFIRI	Y	0	32.6	37.0
P-0001424-T01-IM3	Y	Y	Y	LN	CPT	Y	1	8.4	14.3
P-0001499-T01-IM3	N	Y	Y	lung	CPT/oxali	Y	1	5.1	17.2
P-0001500-T02-IM5	Y	Y	Y	LN	CPT	N	1	33.7	36.5
P-0001693-T02-IM3	Y	Y	N	NONE	FOLFOX	Y	1	14.3	18.8
P-0001702-T01-IM3	Y	Y	Y	LN	FOLFOX	N	1	27.2	32.1
P-0001761-T01-IM3	Y	Y	N	NONE	IRINOTECAN	Y	0	16.3	33.6
P-0001909-T01-IM3	Y	Y	N	NONE	CPT	Y	0	84.9	115.1
P-0001961-T03-IM5	Y	Y	Y	lung	FOLFOX	Y	1	22.5	57.4
P-0001964-T01-IM3	Y	Y	Y	Peritoneum, LN	FOLFIRI	Y	1	26.2	41.0
P-0002070-T01-IM3	Y	Y	N	NONE	FOLFIRI	N	1	12.3	18.7
P-0002095-T01-IM3	Y	Y	Y	Peritoneum, ovary	FOLFIRI	N	0	30.3	36.5
P-0002438-T01-IM3	Y	Y	N	NONE	Capectabine	Y	1	14.0	30.9
P-0002442-T01-IM3	Y	Y	N	NONE	FOLFIRI	Y	0	21.7	30.0
P-0002721-T01-IM3	Y	Y	N	NONE	CPT	N	0	17.6	59.7
P-0002728-T01-IM3	Y	N	N	NONE	CPT/oxali	N	0	24.2	28.2
P-0002752-T01-IM3	Y	Y	N	NONE	5-FU	Y	0	10.6	35.2
P-0002769-T01-IM3	Y	Y	Y	Ovary, LN	FOLFIRI	Y	1	34.6	50.0
P-0002788-T01-IM3	Y	Y	Y	LN	CPT/oxali	N	1	7.1	9.8
P-0002804-T01-IM3	N	Y	N	NONE	FOLFIRI	N	0	29.1	48.5

Supplementary Table 1b continued.

SAMPLE ID	SYNC. DISEASE	ANY CHEMO PRE-PUMP (Y/N)	EXTRAHEP DISEASE PRE-PUMP	EXTRAHEP DISEASE SITE	FIRST SYSTEMIC GIVEN WITH PUMP	LIVER PROG.	VITAL STATUS (1=dead; 0=alive)	LAST FOLLOW UP SINCE FUDR START (months)	LAST FOLLOW UP SINCE DIAGNOSIS (months)
P-0003474-T01-IM5	Y	N	Y	lung	CPT/oxali	Y	1	16.6	18.0
P-0003518-T01-IM5	Y	Y	N	NONE	FOLFIRI	Y	1	36.4	42.0
P-0003789-T01-IM5	Y	Y	N	NONE	CPT	N	0	18.1	29.4
P-0003999-T01-IM5	Y	N	Y	lung	FOLFOX	N	0	23.5	26.2
P-0004018-T01-IM3	Y	Y	Y	Spleen	CPT	Y	1	10.7	25.9
P-0004140-T02-IM5	Y	Y	N	NONE	FOLFIRI	Y	1	38.9	46.3
P-0004251-T01-IM5	Y	Y	Y	LN	FOLFOX	Y	1	5.0	11.5
P-0004322-T01-IM5	Y	Y	N	NONE	5FU/LV	Y	0	32.7	39.2
P-0004323-T01-IM5	Y	Y	Y	Ovary	5-FU + LEUCOVORIN	Y	0	21.2	29.0
P-0004511-T01-IM5	Y	Y	Y	LN	5FU/LV	N	0	22.8	29.3
P-0004626-T01-IM5	Y	Y	N	NONE	FOLFIRI	Y	1	11.5	18.7
P-0004666-T01-IM5	Y	N	N	NONE	CPT/oxali	Y	1	73.8	75.2
P-0004690-T01-IM5	Y	N	N	NONE	CPT/oxali	N	0	24.6	26.0
P-0004698-T01-IM5	Y	N	Y	LN	CPT/oxali	Y	0	24.6	25.5
P-0004744-T01-IM5	Y	Y	Y	LN	CPT/oxali	Y	1	22.0	25.6
P-0004798-T01-IM5	Y	Y	Y	LN	FOLFIRI	Y	1	3.1	12.2
P-0004957-T01-IM5	Y	Y	Y	LN	FOLFIRI	N	1	20.5	38.9
P-0005003-T01-IM5	Y	Y	N	NONE	FOLFIRI	Y	1	5.3	18.2
P-0005132-T01-IM5	Y	N	Y	lung	CPT/oxali	Y	0	62.1	63.2
P-0005164-T01-IM5	Y	N	N	NONE	CPT/oxali	N	0	26.2	27.1
P-0005343-T01-IM5	N	Y	N	NONE	FOLFIRI	N	0	14.4	31.9
P-0005502-T01-IM5	Y	Y	N	NONE	FOLFIRI	N	0	33.3	43.4
P-0005634-T01-IM5	Y	Y	N	NONE	FOLFOX	Y	1	27.0	31.3
P-0005742-T01-IM5	Y	Y	Y	Lung, LN	CPT	N	1	16.9	24.8
P-0005762-T01-IM5	Y	Y	N	NONE	FOLFIRI	Y	0	46.3	53.2

Supplementary Table 1b continued.

SAMPLE ID	SYNC. DISEASE	ANY CHEMO PRE-PUMP (Y/N)	EXTRAHEP DISEASE PRE-PUMP	EXTRAHEP DISEASE SITE	FIRST SYSTEMIC GIVEN WITH PUMP	LIVER PROG.	VITAL STATUS (1=dead; 0=alive)	LAST FOLLOW UP SINCE FUDR START (months)	LAST FOLLOW UP SINCE DIAGNOSIS (months)
P-0005858-T01-IM5	Y	Y	Y	lung	FOLFIRI	Y	0	16.4	28.4
P-0005998-T01-IM5	Y	Y	Y	LN	CPT	Y	1	26.9	31.6
P-0006138-T01-IM5	Y	Y	Y	LN	FOLFIRI	Y	1	8.0	23.6
P-0006271-T01-IM5	Y	Y	Y	LN, lung	Capectabine	N	1	17.8	34.8
P-0006329-T01-IM5	Y	Y	N	NONE	FOLFIRI	N	0	20.1	26.8
P-0006575-T01-IM5	Y	N	Y	LN	CPT/oxali	Y	0	154.9	156.5
P-0006587-T01-IM5	Y	Y	Y	LN	FOLFOX	N	0	17.2	22.2
P-0006608-T01-IM5	Y	Y	N	NONE	FOLFIRI	Y	0	47.2	62.6
P-0006616-T01-IM5	Y	N	Y	Peritoneum	FOLFOX	N	1	12.6	14.3
P-0006657-T01-IM5	Y	Y	N	NONE	CPT/oxali	Y	1	24.4	34.3
P-0006658-T01-IM5	Y	Y	N	NONE	FOLFIRI	N	0	90.2	94.6
P-0006683-T01-IM5	Y	Y	N	NONE	FOLFIRI	Y	1	24.4	28.5
P-0006685-T01-IM5	Y	Y	Y	LN	CPT	Y	1	15.6	36.4
P-0006693-T01-IM5	Y	Y	N	NONE	5FU/LV	N	0	6.7	23.2
P-0006707-T01-IM5	Y	N	N	NONE	CPT/oxali	Y	0	42.5	43.4
P-0006708-T01-IM5	Y	Y	N	NONE	FOLFIRI	Y	1	25.6	31.4
P-0006715-T01-IM5	Y	Y	Y	LN	FOLFIRI	Y	0	30.6	37.9
P-0006721-T01-IM5	N	Y	N	NONE	FOLFIRI	N	0	19.6	60.1
P-0006729-T01-IM5	Y	Y	N	NONE	FOLFOX	N	0	24.6	30.3
P-0006743-T01-IM5	Y	Y	Y	lung	FOLFIRI	Y	0	33.0	42.8
P-0006750-T01-IM5	Y	Y	N	NONE	CPT	Y	0	16.8	38.4
P-0006794-T01-IM5	Y	N	N	NONE	CPT/oxali	N	0	71.7	72.8
P-0006835-T01-IM5	Y	Y	N	NONE	FOLFIRI	Y	1	35.4	46.9
P-0006853-T01-IM5	Y	Y	N	NONE	5FU/LV	N	1	26.7	30.8
P-0006864-T01-IM5	Y	Y	N	NONE	FOLFIRI	Y	0	15.2	24.0
P-0006936-T01-IM5	Y	Y	N	NONE	FOLFIRI	Y	1	90.3	109.0

Supplementary Table 1b continued.

SAMPLE ID	SYNC. DISEASE	ANY CHEMO PRE-PUMP (Y/N)	EXTRAHEP DISEASE PRE-PUMP	EXTRAHEP DISEASE SITE	FIRST SYSTEMIC GIVEN WITH PUMP	LIVER PROG.	VITAL STATUS (1=dead; 0=alive)	LAST FOLLOW UP SINCE FUDR START (months)	LAST FOLLOW UP SINCE DIAGNOSIS (months)
P-0006978-T01-IM5	Y	Y	N	NONE	CPT/oxali	Y	0	63.6	68.0
P-0006983-T01-IM5	Y	Y	N	NONE	CPT/oxali	Y	1	41.9	49.2
P-0006999-T02-IM5	Y	Y	Y	LN	CPT	Y	1	176.4	187.4
P-0007012-T01-IM5	Y	Y	Y	LN	FOLFIRI	Y	1	22.1	28.3
P-0007039-T01-IM5	Y	Y	Y	Ovary	FOLFOX	Y	1	44.8	48.8
P-0007120-T01-IM5	N	Y	Y	LN	FOLFIRI	N	0	14.3	50.3
P-0007132-T01-IM5	N	Y	N	NONE	FOLFIRI	Y	0	102.9	133.8
P-0007135-T01-IM5	Y	Y	N	NONE	FOLFIRI	Y	0	56.8	61.1
P-0007140-T01-IM5	N	Y	N	NONE	FOLFIRI	Y	0	14.5	41.1
P-0007185-T01-IM5	Y	Y	N	NONE	FOLFIRI	Y	0	62.7	71.6
P-0007272-T01-IM5	Y	Y	N	NONE	CPT/oxali	N	0	24.0	27.5
P-0007279-T01-IM5	Y	Y	N	NONE	CPT	Y	0	50.2	59.1
P-0007287-T01-IM5	Y	N	N	NONE	CPT/oxali	Y	0	59.0	60.8
P-0007334-T01-IM5	Y	Y	Y	LN	FOLFIRI	N	0	30.0	36.7
P-0007365-T01-IM5	Y	Y	N	NONE	CPT	Y	1	79.9	91.8
P-0007405-T01-IM5	Y	N	N	NONE	CPT/oxali	Y	0	56.4	58.2
P-0007489-T01-IM5	Y	N	N	NONE	CPT/oxali	Y	0	161.1	162.3
P-0007586-T01-IM5	Y	Y	Y	LN	FOLFOX	N	0	61.9	65.5
P-0007591-T01-IM5	Y	N	N	NONE	CPT/oxali	Y	1	76.8	78.8
P-0007683-T01-IM5	Y	N	N	NONE	CPT/oxali	N	0	19.4	21.4
P-0007739-T01-IM5	Y	N	N	NONE	CPT	N	0	166.0	166.9
P-0007771-T01-IM5	Y	Y	N	NONE	FOLFIRI	Y	1	116.3	131.8
P-0007887-T01-IM5	Y	Y	N	NONE	FOLFOX	Y	0	116.2	120.4
P-0008022-T02-IM5	Y	Y	Y	LN	FOLFIRI	N	1	12.0	31.1
P-0008031-T01-IM5	Y	Y	N	NONE	FOLFIRI	Y	0	14.2	21.1
P-0008042-T01-IM5	Y	Y	N	NONE	FOLFIRI	Y	0	38.9	43.8

Supplementary Table 1b continued.

SAMPLE ID	SYNC. DISEASE	ANY CHEMO PRE-PUMP (Y/N)	EXTRAHEP DISEASE PRE-PUMP	EXTRAHEP DISEASE SITE	FIRST SYSTEMIC GIVEN WITH PUMP	LIVER PROG.	VITAL STATUS (1=dead; 0=alive)	LAST FOLLOW UP SINCE FUDR START (months)	LAST FOLLOW UP SINCE DIAGNOSIS (months)
P-0008075-T01-IM5	Y	Y	N	NONE	CPT/oxali	Y	1	46.6	51.3
P-0008079-T01-IM5	N	Y	Y	Omentum	FOLFIRI	N	0	71.3	108.2
P-0008083-T01-IM5	Y	N	N	NONE	FOLFOX	Y	0	58.2	59.6
P-0008094-T01-IM5	Y	Y	N	NONE	FOLFIRI	Y	1	96.5	105.9
P-0008267-T01-IM5	Y	Y	N	NONE	FOLFOX	N	0	18.1	22.2
P-0008483-T01-IM5	Y	N	Y	Peritoneum	FOLFOX	N	0	16.1	18.3
P-0008534-T01-IM5	Y	Y	N	NONE	FOLFIRI	N	0	19.1	23.3
P-0008584-T01-IM5	Y	N	N	NONE	OXALIPLATIN + IRINOTECAN	Y	0	115.3	116.4
P-0008600-T01-IM5	N	Y	N	NONE	FOLFIRI	Y	0	19.6	150.7
P-0009377-T01-IM5	Y	N	N	NONE	OXALIPLATIN + IRINOTECAN	NA	0	97.2	99.6
P-0009488-T01-IM5	Y	Y	N	NONE	FOLFIRI	Y	1	13.7	17.7
P-0009650-T01-IM5	Y	Y	N	NONE	5-FU	Y	0	17.7	24.1
P-0009972-T01-IM5	Y	Y	N	NONE	FOLFIRI	Y	0	11.1	15.8
P-0010014-T01-IM5	Y	Y	N	NONE	IRINOTECAN	N	0	96.5	125.2
P-0010180-T01-IM5	Y	N	N	NONE	OXALIPLATIN + IRINOTECAN	Y	0	92.3	93.2
P-0010364-T01-IM5	N	Y	N	NONE	FOLFIRI	Y	0	16.3	47.8
P-0010367-T01-IM5	Y	Y	N	NONE	IRINOTECAN	Y	0	15.2	35.1
P-0010406-T01-IM5	Y	Y	N	NONE	FOLFOX	N	0	16.6	21.8
P-0011296-T01-IM5	Y	Y	N	NONE	IRINOTECAN	N	0	95.0	107.3
P-0011306-T01-IM5	Y	Y	N	NONE	FOLFOX	N	0	11.3	18.9
P-0011455-T01-IM5	Y	Y	N	NONE	NONE	Y	0	12.5	49.7
P-0011520-T01-IM5	Y	Y	N	NONE	5-FU	N	0	12.0	23.0
P-0012281-T01-IM5	Y	Y	Y	lung	FOLFOX	Y	0	50.2	57.1
P-0012426-T01-IM5	Y	Y	N	NONE	IRINOTECAN	N	0	58.3	65.5

Supplementary Table 1b continued.

SAMPLE ID	SYNC. DISEASE	ANY CHEMO PRE-PUMP (Y/N)	EXTRAHEP DISEASE PRE-PUMP	EXTRAHEP DISEASE SITE	FIRST SYSTEMIC GIVEN WITH PUMP	LIVER PROG.	VITAL STATUS (1=dead; 0=alive)	LAST FOLLOW UP SINCE FUDR START (months)	LAST FOLLOW UP SINCE DIAGNOSIS (months)
P-0012625-T01-IM5	N	Y	N	NONE	FOLFIRI	Y	1	14.8	41.3
P-0012722-T01-IM5	Y	Y	N	NONE	FOLFOX	Y	0	9.4	15.8
P-0012823-T01-IM5	Y	Y	N	NONE	NONE	N	0	0.9	14.2
P-0013201-T01-IM5	N	Y	N	NONE	FOLFIRI	N	0	12.7	39.1
P-0013881-T01-IM5	Y	Y	N	NONE	IRINOTECAN	N	0	8.5	17.3
P-0013982-T01-IM5	Y	Y	N	NONE	FOLFIRI	N	0	1.7	12.8
P-0012625-T01-IM5	N	Y	N	NONE	FOLFIRI	Y	1	14.8	41.3
P-0012722-T01-IM5	Y	Y	N	NONE	FOLFOX	Y	0	9.4	15.8
P-0012823-T01-IM5	Y	Y	N	NONE	NONE	N	0	0.9	14.2
P-0013201-T01-IM5	N	Y	N	NONE	FOLFIRI	N	0	12.7	39.1
P-0013881-T01-IM5	Y	Y	N	NONE	IRINOTECAN	N	0	8.5	17.3
P-0013982-T01-IM5	Y	Y	N	NONE	FOLFIRI	N	0	1.7	12.8

Supplementary Table 2: Demographic and clinical characteristics of the validation cohort.

	OVERALL (n=93)	Adj (n=43)	Met (n=50)
**Sex**			
Male	53 (57)	26 (60)	27 (54)
Female	40 (43)	17 (40)	23 (46)
**Age, median (range, yrs)**			
24-50, n (%)	38 (41)	16 (37)	22 (44)
51-74, n (%)	53 (57)	26 (60)	27 (54)
>=75, n (%)	2 (2)	1 (2)	1 (2)
**Site of Primary, n (%)**			
Left colon	18 (19)	9 (21)	9 (18)
Right colon	52 (56)	23 (53)	29 (58)
Rectum	23 (25)	11 (26)	12 (24)
**Lymph node positive primary, n (%)**			
Yes	65 (70)	24 (56)	41 (82)
No	28 (30)	19 (44)	9 (18)
**Synchronous disease, n (%)**			
Yes	77 (83)	32 (74)	45 (90)
No	16 (17)	11 (26)	5 (10)
**Systemic chemotherapy prior to HAI, n (%)**			
Yes	77 (83)	35 (81)	42 (84)
No	16 (17)	8 (19)	8 (16)
**First systemic regimen given with HAI, n (%)**			
5-FU/LV or Capecitabine	17 (18)	12 (28)	5 (10)
Irinotecan alone	7 (8)	4 (9)	3 (6)
Oxaliplatin/Irinotecan	5 (5)	0 (0)	5 (10)
FOLFIRI	32 (34)	9 (21)	23 (46)
FOLFOX	15 (16)	8 (19)	7 (14)
FOLFIRI + Anti-EGFR	4 (4)	3 (7)	1 (2)
FOLFIRI or FOLFOX + Anti-VEGF	4 (4)	4 (9)	0 (0)
Mitomycin +/- 5-FU + Irinotecan	4 (4)	0 (0)	4 (8)
None[§]	5 (5)	3 (7)	2 (4)
**Extrahepatic Disease before HAI, n (%)**			
Yes	31 (33)	8 (19)	23 (46)
No	62 (67)	35 (81)	27 (54)
**Site of Extrahepatic Disease before HAI[b], n (%)**			
Lung	16 (17)	5 (12)	11 (22)
Lymph node(s)	9 (10)	1 (2)	8 (16)
Peritoneum	1 (1)	0 (0)	1 (2)
Ovary	2 (2)	1 (2)	1 (2)
Other	3 (3)	1 (2)	2 (4)
**Hepatic Progression of Disease (POD), n (%)**			
Yes	66 (71)	19 (44)	47 (94)
No	27 (29)	24 (56)	3 (6)

For the validation cohort, patient diagnosis dates ranged from November 2002 to May 2018. Tumors were sequenced between February 2015 and November 2018.

Supplementary Table 3a: Adj cohort Gene Level, *Ras/B-Raf* wild type

Gene	#WT	#ALT	WT Median Survival (mon)	ALT Median Survival (mon)	WT 2-yr Survival	ALT 2-yr Survival	WT 5-yr Survival	ALT 5-yr Survival	P-value	Q-value
EGFR	109	6	NA	47.67	74%	83%	36%	0%	0	0.00004
NCOR1	112	3	NA	66.74	74%	100%	33%	67%	0.00396	0.07914
PIK3CA	105	10	NA	146.27	75%	70%	35%	20%	0.10997	0.89262
SMAD2	111	4	NA	NA	74%	100%	32%	75%	0.32862	0.89262
TP53	8	107	54.12	NA	88%	74%	12%	36%	0.38082	0.89262
SMAD3	111	4	NA	NA	74%	100%	32%	75%	0.39136	0.89262
ERBB2	106	9	NA	NA	76%	56%	35%	22%	0.45625	0.89262
FBXW7	108	7	NA	146.27	75%	71%	34%	29%	0.45737	0.89262
PTEN	110	5	NA	NA	74%	100%	34%	40%	0.47975	0.89262
ARID1A	108	7	NA	NA	76%	57%	33%	43%	0.49696	0.89262
RASA1	112	3	NA	NA	74%	100%	34%	33%	0.50015	0.89262
MYC	110	5	NA	NA	75%	80%	33%	60%	0.5406	0.89262
FGFR1	110	5	NA	NA	74%	100%	33%	60%	0.54902	0.89262
AURKA	107	8	NA	NA	75%	75%	33%	50%	0.56822	0.89262
TET1	112	3	NA	NA	74%	100%	34%	33%	0.59071	0.89262
SOX9	104	11	NA	NA	74%	82%	35%	27%	0.59135	0.89262
FAT1	110	5	NA	NA	74%	100%	35%	20%	0.59159	0.89262
SMAD4	103	12	NA	NA	75%	75%	34%	33%	0.60719	0.89262
ASXL1	113	2	NA	NA	74%	100%	34%	50%	0.6121	0.89262
FOXP1	112	3	NA	NA	74%	100%	34%	33%	0.61374	0.89262
ERBB3	111	4	NA	NA	75%	75%	34%	25%	0.62194	0.89262
TCF7L2	107	8	NA	NA	75%	75%	34%	38%	0.63525	0.89262
KMT2D	112	3	NA	NA	74%	100%	35%	0%	0.63566	0.89262
RNF43	112	3	NA	NA	74%	100%	34%	33%	0.63566	0.89262

Supplementary Table 3a continued.

Gene	#WT	#ALT	WT Median Survival (mon)	ALT Median Survival (mon)	WT 2-yr Survival	ALT 2-yr Survival	WT 5-yr Survival	ALT 5-yr Survival	P-value	Q-value
CDK8	109	6	NA	NA	75%	67%	35%	17%	0.65465	0.89262
FLT3	108	7	NA	NA	74%	86%	35%	14%	0.65465	0.89262
RAD50	113	2	NA	NA	74%	100%	34%	50%	0.66273	0.89262
AXIN2	112	3	NA	NA	75%	67%	35%	0%	0.7232	0.89262
CIC	113	2	NA	NA	74%	100%	35%	0%	0.7232	0.89262
KMT2A	112	3	NA	NA	75%	67%	35%	0%	0.7232	0.89262
MAP2K4	112	3	NA	NA	75%	67%	34%	33%	0.7241	0.89262
MAP3K1	113	2	NA	NA	74%	100%	35%	0%	0.74153	0.89262
PBRM1	113	2	NA	NA	75%	50%	35%	0%	0.75878	0.89262
TERT	114	1	NA	NA	75%	100%	34%	0%	0.75878	0.89262
BCL2L1	101	14	NA	NA	74%	79%	32%	50%	0.81698	0.89262
DNMT3B	102	13	NA	NA	74%	85%	31%	54%	0.81698	0.89262
APC	14	101	NA	NA	86%	73%	29%	35%	0.842	0.89262
SRC	103	12	NA	NA	75%	75%	31%	58%	0.84798	0.89262
AMER1	113	2	NA	NA	75%	50%	35%	0%	1	1
ATM	114	1	NA	NA	75%	0%	34%	0%	1	1

Supplementary Table 3b: Adj cohort Gene Level, *Ras/B-Raf* altered

Gene	#WT	#ALT	WT Median Survival (mon)	ALT Median Survival (mon)	WT 2-yr Survival	ALT 2-yr Survival	WT 5-yr Survival	ALT 5-yr Survival	P-value	Q-value
AXIN2	73	1	NA	27.75	75%	100%	27%	0%	0	0.00001
TERT	72	2	NA	19.1	76%	50%	28%	0%	0.00002	0.00042
TET1	73	1	NA	34.36	75%	100%	27%	0%	0.00007	0.00084
CDK8	71	3	NA	42.33	76%	67%	28%	0%	0.00042	0.00367
SMAD4	63	11	NA	66.08	78%	64%	30%	9%	0.00197	0.01378
FLT3	71	3	NA	50.3	77%	33%	28%	0%	0.0658	0.38273
CTNNB1	71	3	NA	34.36	76%	67%	27%	33%	0.07655	0.38273
TP53	25	49	NA	77.56	76%	76%	24%	29%	0.26344	0.82636
ASXL1	71	3	NA	NA	76%	67%	25%	67%	0.31823	0.82636
CIC	72	2	NA	NA	75%	100%	25%	100%	0.31823	0.82636
SOX9	64	10	NA	57.27	80%	50%	30%	10%	0.34487	0.82636
FAT1	72	2	NA	NA	75%	100%	26%	50%	0.39554	0.82636
FBXW7	71	3	NA	NA	75%	100%	27%	33%	0.41711	0.82636
ATM	71	3	NA	NA	76%	67%	27%	33%	0.42546	0.82636
AMER1	70	4	NA	NA	77%	50%	27%	25%	0.43979	0.82636
BCL2L1	73	1	NA	NA	75%	100%	26%	100%	0.44636	0.82636
DNMT3B	73	1	NA	NA	75%	100%	26%	100%	0.44636	0.82636
SRC	73	1	NA	NA	75%	100%	26%	100%	0.44636	0.82636
SMAD2	67	7	NA	NA	78%	57%	30%	0%	0.44859	0.82636
KMT2D	73	1	NA	NA	75%	100%	27%	0%	0.59368	0.92454
RAD50	73	1	NA	NA	75%	100%	27%	0%	0.59368	0.92454
APC	16	58	NA	NA	69%	78%	19%	29%	0.64106	0.92454
RNF43	72	2	NA	NA	76%	50%	28%	0%	0.68307	0.92454
SMAD3	71	3	NA	NA	77%	33%	28%	0%	0.72494	0.92454

Supplementary Table 3b continued.

Gene	#WT	#ALT	WT Median Survival (mon)	ALT Median Survival (mon)	WT 2-yr Survival	ALT 2-yr Survival	WT 5-yr Survival	ALT 5-yr Survival	P-value	Q-value
PTEN	69	5	NA	77.56	75%	80%	26%	40%	0.74533	0.92454
ERBB2	73	1	NA	NA	75%	100%	27%	0%	0.77478	0.92454
TCF7L2	68	6	NA	44.78	76%	67%	28%	17%	0.77805	0.92454
MAP3K1	71	3	NA	NA	77%	33%	28%	0%	0.8216	0.92454
MYC	73	1	NA	NA	75%	100%	27%	0%	0.84871	0.92454
ARID1A	73	1	NA	NA	77%	0%	27%	0%	0.89812	0.92454
ERBB3	73	1	NA	NA	77%	0%	27%	0%	0.89812	0.92454
MAP2K4	73	1	NA	NA	75%	100%	27%	0%	0.89812	0.92454
PBRM1	72	2	NA	NA	76%	50%	28%	0%	0.89812	0.92454
FGFR1	73	1	NA	NA	77%	0%	27%	0%	0.89812	0.92454
PIK3CA	56	18	NA	NA	75%	78%	27%	28%	0.94667	0.94667

Supplementary Table 3c. Adj cohort power level, *Ras/B-Raf* wild type

Pathway	#WT	#ALT	WT Median Survival (mon)	ALT Median Survival (Mon)	WT 2-yr Survival	ALT 2-yr Survival	WT 5-yr Survival	ALT 5-yr Survival	P-value	Q-value
Cell_Cycle_pthwy	106	9	NA	73.18	73%	100%	34%	33%	0.00191	0.01528
NOTCH_pthwy	101	14	NA	146.27	74%	79%	33%	43%	0.00502	0.0201
TP53_pthwy	8	107	54.12	NA	88%	74%	12%	36%	0.38082	0.72202
HIPPO_pthwy	109	6	NA	NA	73%	100%	34%	33%	0.42942	0.72202
MYC_pthwy	109	6	NA	NA	74%	83%	32%	67%	0.45821	0.72202
TGF_Beta_pthwy	97	18	NA	NA	73%	83%	31%	50%	0.54151	0.72202
WNT_pthwy	13	102	NA	NA	85%	74%	31%	34%	0.74618	0.85278
PI3K_pthwy	95	20	NA	NA	74%	80%	34%	35%	0.89865	0.89865

Supplementary Table 3d. Adj cohort power level, *Ras/B-Raf* altered

Pathway	#WT	#ALT	WT Median Survival (mon)	ALT Median Survival (Mon)	WT 2-yr Survival	ALT 2-yr Survival	WT 5-yr Survival	ALT 5-yr Survival	P-value	Q-value
TGF_Beta_pthwy	58	16	NA	66.08	79%	62%	33%	6%	0.02152	0.17218
WNT_pthwy	10	64	NA	NA	90%	73%	30%	27%	0.08636	0.34545
HIPPO_pthwy	72	2	NA	NA	75%	100%	26%	50%	0.39554	0.66572
NOTCH_pthwy	70	4	NA	NA	76%	75%	27%	25%	0.41079	0.66572
TP53_pthwy	22	52	NA	NA	77%	75%	23%	29%	0.41608	0.66572
PI3K_pthwy	48	26	NA	NA	75%	77%	27%	27%	0.83575	0.96995
MYC_pthwy	73	1	NA	NA	75%	100%	27%	0%	0.84871	0.96995
Cell_Cycle_pthwy	73	1	NA	NA	77%	0%	27%	0%	1	1

Supplementary Table 3e: Met cohort Gene Level, *Ras/B-Raf* wild type

Gene	#WT	#ALT	WT Median Survival (mon)	ALT Median Survival (mon)	WT 2-yr Survival	ALT 2-yr Survival	WT 5-yr Survival	ALT 5-yr Survival	P-value	Q-value
ARID1A	96	3	116.25	11.97	53%	0%	25%	0%	0	0
SMAD3	98	1	116.25	15.65	52%	0%	24%	0%	0.01413	0.25426
PTEN	95	4	116.25	29.33	52%	50%	24%	25%	0.07389	0.80415
SMAD2	96	3	116.25	22.09	52%	33%	24%	33%	0.08935	0.80415
SMARCA4	97	2	96.46	NA	51%	100%	24%	50%	0.25837	0.86295
BCL2L1	90	9	96.46	NA	52%	44%	23%	33%	0.26995	0.86295
FGFR1	95	4	116.25	NA	51%	75%	24%	25%	0.31268	0.86295
ERBB2	92	7	116.25	73.81	49%	86%	23%	43%	0.33595	0.86295
BCOR	98	1	116.25	NA	51%	100%	23%	100%	0.35064	0.86295
SMAD4	85	14	96.46	116.25	52%	50%	22%	36%	0.39346	0.86295
AURKA	96	3	116.25	11.97	52%	33%	25%	0%	0.41814	0.86295
APC	18	81	96.46	116.25	72%	47%	33%	22%	0.4319	0.86295
MGA	96	3	116.25	NA	52%	33%	24%	33%	0.43286	0.86295
FBXW7	95	4	116.25	NA	53%	25%	25%	0%	0.43699	0.86295
RBM10	97	2	116.25	NA	52%	50%	24%	50%	0.46241	0.86295
ERBB3	98	1	116.25	NA	51%	100%	24%	0%	0.53242	0.86295
TGFBR2	98	1	116.25	NA	51%	100%	23%	100%	0.53242	0.86295
MAP2K4	96	3	116.25	NA	52%	33%	25%	0%	0.54303	0.86295
PIK3R1	93	6	116.25	NA	53%	33%	25%	17%	0.55073	0.86295
PIK3CA	90	9	116.25	73.81	50%	67%	24%	22%	0.61684	0.86295
TCF7L2	90	9	116.25	NA	53%	33%	26%	11%	0.62008	0.86295
EGFR	95	4	116.25	73.81	49%	100%	23%	50%	0.6266	0.86295
MAPK1	97	2	116.25	30.61	51%	100%	25%	0%	0.62905	0.86295
CDK8	98	1	116.25	NA	51%	100%	24%	0%	0.63662	0.86295

Supplementary Table 3e continued.

Gene	#WT	#ALT	WT Median Survival (mon)	ALT Median Survival (mon)	WT 2-yr Survival	ALT 2-yr Survival	WT 5-yr Survival	ALT 5-yr Survival	P-value	Q-value
FLT3	98	1	116.25	NA	51%	100%	24%	0%	0.63662	0.86295
SRC	92	7	116.25	NA	53%	29%	25%	14%	0.66673	0.86295
SOX9	91	8	96.46	116.25	51%	62%	24%	25%	0.67073	0.86295
MYC	88	11	116.25	176.42	51%	55%	23%	36%	0.68609	0.86295
CCND2	97	2	116.25	79.89	52%	50%	24%	50%	0.71614	0.86295
ASXL1	98	1	116.25	NA	52%	0%	24%	0%	0.71913	0.86295
MAP3K1	98	1	116.25	NA	52%	0%	24%	0%	0.7502	0.87121
DNMT3B	94	5	116.25	NA	52%	40%	24%	20%	0.77733	0.87449
TP53	8	91	96.46	116.25	50%	52%	38%	23%	0.8183	0.89269
MET	96	3	116.25	38.79	51%	67%	24%	33%	0.87704	0.92863

Supplementary Table 3f. Met cohort Gene Level, *Ras/B-Raf* altered

Gene	#WT	#ALT	WT Median Survival (mon)	ALT Median Survival (mon)	WT 2-yr Survival	ALT 2-yr Survival	WT 5-yr Survival	ALT 5-yr Survival	P-value	Q-value
SMARCA4	81	1	26.96	7.07	37%	0%	5%	0%	0.00003	0.0011
BCOR	80	2	26.96	3.06	38%	0%	5%	0%	0.00271	0.04877
PIK3R1	80	2	27.22	10.93	38%	0%	5%	0%	0.00444	0.05334
MAPK1	81	1	26.96	10.16	37%	0%	5%	0%	0.00694	0.06243
MAP2K4	81	1	26.96	10.65	37%	0%	5%	0%	0.02118	0.12707
CCND2	81	1	26.96	10.65	37%	0%	5%	0%	0.02118	0.12707
TP53	23	59	58.19	26.2	48%	32%	9%	3%	0.03048	0.15678
PTEN	77	5	26.96	NA	39%	0%	5%	0%	0.07905	0.35571
FLT3	77	5	26.86	NA	36%	40%	5%	0%	0.10798	0.43191
BCL2L1	81	1	26.96	NA	36%	100%	4%	100%	0.14436	0.51968
SMAD4	67	15	26.7	58.19	33%	53%	3%	13%	0.19955	0.65306
CDK8	79	3	26.96	NA	37%	33%	5%	0%	0.226	0.678
MYC	79	3	27.22	26.2	37%	33%	5%	0%	0.30765	0.75049
FBXW7	72	10	26.96	33.73	38%	30%	6%	0%	0.32137	0.75049
AMER1	79	3	26.96	NA	38%	0%	5%	0%	0.32622	0.75049
MAP3K1	80	2	26.96	NA	36%	50%	5%	0%	0.33355	0.75049
RBM10	79	3	26.96	NA	38%	0%	5%	0%	0.38874	0.82321
TCF7L2	79	3	26.96	24.36	35%	67%	4%	33%	0.44131	0.85819
ERBB2	76	6	27.22	22.75	39%	0%	5%	0%	0.45293	0.85819
ASXL1	80	2	27.22	20.52	38%	0%	5%	0%	0.49189	0.87657
ERBB3	80	2	26.96	28.24	36%	50%	5%	0%	0.51133	0.87657
SOX9	78	4	26.86	27.22	36%	50%	5%	0%	0.53621	0.87744
MET	81	1	27.22	26.2	36%	100%	5%	0%	0.56391	0.88265
SMAD3	78	4	26.96	NA	37%	25%	5%	0%	0.63635	0.9115

Supplementary Table 3f continued.

Gene	#WT	#ALT	WT Median Survival (mon)	ALT Median Survival (mon)	WT 2-yr Survival	ALT 2-yr Survival	WT 5-yr Survival	ALT 5-yr Survival	P-value	Q-value
TGFBR2	80	2	26.96	16.9	36%	50%	5%	0%	0.64939	0.9115
FGFR1	81	1	26.96	NA	37%	0%	5%	0%	0.6583	0.9115
PIK3CA	64	18	26.86	58.19	38%	33%	6%	0%	0.74313	0.95527
SMAD2	79	3	26.96	16.57	38%	0%	5%	0%	0.75224	0.95527
TERT	79	3	26.96	22.75	38%	0%	5%	0%	0.76952	0.95527
ARID1A	81	1	26.96	NA	37%	0%	5%	0%	0.91042	1
APC	14	68	38.89	26.86	36%	37%	14%	3%	0.93169	1

Supplementary Table 3g. Met cohort power level, *Ras/B-Raf* wild type

Pathway	#WT	#ALT	WT Median Survival (mon)	ALT Median Survival (Mon)	WT 2-yr Survival	ALT 2-yr Survival	WT 5-yr Survival	ALT 5-yr Survival	P-value	Q-value
PI3K_pthwy	79	20	176.42	73.81	51%	55%	24%	25%	0.08147	0.57506
TGF_Beta_pthwy	82	17	176.42	116.25	52%	47%	22%	35%	0.14376	0.57506
HIPPO_pthwy	97	2	176.42	65.11	52%	50%	24%	50%	0.24051	0.58944
NOTCH_pthwy	93	6	116.25	NA	54%	17%	26%	0%	0.34897	0.58944
WNT_pthwy	16	83	79.89	116.25	69%	48%	31%	23%	0.3684	0.58944
MYC_pthwy	85	14	116.25	176.42	52%	50%	22%	36%	0.48723	0.59801
Cell_Cycle_pthwy	94	5	116.25	79.89	51%	60%	22%	60%	0.52326	0.59801
TP53_pthwy	8	91	96.46	116.25	50%	52%	38%	23%	0.8183	0.8183

Supplementary Table 3h. Met cohort power level, *Ras/B-Raf* altered

Pathway	#WT	#ALT	WT Median Survival (mon)	ALT Median Survival (Mon)	WT 2-yr Survival	ALT 2-yr Survival	WT 5-yr Survival	ALT 5-yr Survival	P-value	Q-value
Cell_Cycle_pthwy	80	2	27.22	8.91	38%	0%	5%	0%	0.00007	0.00054
TP53_pthwy	23	59	58.19	26.2	48%	32%	9%	3%	0.03048	0.12194
TGF_Beta_pthwy	59	23	26.86	58.19	34%	43%	3%	9%	0.18981	0.50616
MYC_pthwy	79	3	27.22	26.2	37%	33%	5%	0%	0.30765	0.51419
NOTCH_pthwy	72	10	26.96	33.73	38%	30%	6%	0%	0.32137	0.51419
PI3K_pthwy	58	24	26.96	27.22	41%	25%	7%	0%	0.52072	0.69429
WNT_pthwy	13	69	38.89	26.96	31%	38%	8%	4%	0.65595	0.74965
